# Iron homeostasis in the absence of ferricrocin and its consequences in fungal development and insect virulence in *Beauveria bassiana*

**DOI:** 10.1038/s41598-021-99030-4

**Published:** 2021-10-04

**Authors:** Jiraporn Jirakkakul, Nuchnudda Wichienchote, Somsak Likhitrattanapisal, Supawadee Ingsriswang, Thippawan Yoocha, Sithichoke Tangphatsornruang, Rudsamee Wasuwan, Supapon Cheevadhanarak, Morakot Tanticharoen, Alongkorn Amnuaykanjanasin

**Affiliations:** 1 Pilot Plant Development and Training Institute, King Mongkut’s University of Technology Thonburi, Bang Khun Thian, Bangkok, 10150 Thailand; 2 National Center for Genetic Engineering and Biotechnology (BIOTEC), National Science and Technology Development Agency, 113 Thailand Science Park, Phahonyothin Rd., Tambon Khlong Nueng, Amphoe Khlong Luang, Pathum Thani 12120 Thailand; 3 National Omics Center, National Science and Technology Development Agency, 111 Thailand Science Park, Phahonyothin Rd., Khlong Nueng, Khlong Luang, Pathum Thani 12120 Thailand; 4 School of Bioresources and Technology, King Mongkut’s University of Technology Thonburi, Bang Khun Thian, Bangkok, 10150 Thailand

**Keywords:** Biochemistry, Cell biology, Genetics, Microbiology, Molecular biology, Physiology

## Abstract

The putative ferricrocin synthetase gene *ferS* in the fungal entomopathogen *Beauveria bassiana* BCC 2660 was identified and characterized. The 14,445-bp *ferS* encodes a multimodular nonribosomal siderophore synthetase tightly clustered with *Fusarium graminearum* ferricrocin synthetase. Functional analysis of this gene was performed by disruption with the *bar* cassette. Δ*ferS* mutants were verified by Southern and PCR analyses. HPLC and TLC analyses of crude extracts indicated that biosynthesis of ferricrocin was abolished in Δ*ferS*. Insect bioassays surprisingly indicated that Δ*ferS* killed the *Spodoptera exigua* larvae faster (LT_50_ 59 h) than wild type (66 h). Growth and developmental assays of the mutant and wild type demonstrated that Δ*ferS* had a significant increase in germination under iron depletion and radial growth and a decrease in conidiation. Mitotracker staining showed that the mitochondrial activity was enriched in Δ*ferS* under both iron excess and iron depletion. Comparative transcriptomes between wild type and Δ*ferS* indicated that the mutant was increased in the expression of eight cytochrome P450 genes and those in iron homeostasis, ferroptosis, oxidative stress response, ergosterol biosynthesis, and TCA cycle, compared to wild type. Our data suggested that Δ*ferS* sensed the iron excess and the oxidative stress and, in turn, was up-regulated in the antioxidant-related genes and those in ergosterol biosynthesis and TCA cycle. These increased biological pathways help Δ*ferS* grow and germinate faster than the wild type and caused higher insect mortality than the wild type in the early phase of infection.

## Introduction

Iron homeostasis is essential in nearly all living forms, from mammals to microbes. It maintains a balance between the iron supply and the prevention of cellular toxicity due to iron overload^1,2^. In fungi and bacteria, small iron-chelating compounds called ‘siderophores’ and transporters mediate iron-associated mechanisms, similar to those found in humans and mammals^3–5^. Insect-pathogenic fungi are microbes used for the biological control of agricultural insect pests. *Beauveria bassiana* is a widely-regarded biological control agent used commercially in several countries, including Thailand. As an entomopathogen, iron is indispensable for the initiation and establishment of *B. bassiana* in insect hosts^6^. Fungal cells primarily utilized small molecules "siderophores" to sequestor iron from the environment, thus being an extracellular siderophore, and to store and distribute iron in the cell, being an intracellular siderophore. Ferricrocin is a common intracellular siderophores in several fungi, including *Aspergillus fumigatus*^7^, *A. nidulans*^8^, and *Metarhizium robertsii*^9^.

A major group of enzymes that synthesize siderophores belong to the family of nonribosomal peptide synthetases (NRPSs). Several siderophores are synthesized by these multimodular NRPSs. Each NRPS module has an adenylation domain (A), thiolation domain (T), and condensation domain (C), thus activating and incorporating one amino acid. The phylogeny of ferrichrome synthetases is based on their modular evolution, and their domain architecture divides these enzymes into two lineages, NPS1/SidC lineage, and NPS2 lineage. Most ferrichromes consist of three *N*^5^-acyl-*N*^5^-hydroxy-L-ornithine (AHO) and three amino acids. One amino acid is always a glycine, and the remaining two can be a combination of alanine, serine, or glycine. For example, ferrichrome A consists of three AHOs, one glycine, and two serines. Ferricrocin consists of three AHOs, with two glycines and one serine^10^. Although several fungal NRPSs associated with intracellular siderophore biosynthesis have been studied, there are distinct roles for the intracellular siderophores of different fungi, particularly among fungal pathogens. For example, the ferricrocin synthesis gene *ssm1* is involved in intracellular siderophore production in the phytopathogenic fungus *Magnaporthe grisea*. It contributes to the plant infection process, including the formation of a penetration peg. The *ssm1* mutation affected fungal pathogenicity in rice^11^. In contrast, the disruption of ferrichrome synthetase gene *sid1* (Δ*sid1*) in plant pathogenic fungus *Ustilago maydis* did not affect its phytopathogenicity^12^.

Previously, *sidC1* that encodes a monomodular nonribosomal peptide synthetase has been knocked down by RNA silencing in *B. bassiana* BCC 2660^13^. In this study, we completely knocked out the ferricrocin synthetase gene *ferS* by targeted disruption. We performed comprehensive studies of Δ*ferS* compared with *B. bassiana* wild type. The biosynthesis of ferricrocin has been abolished in Δ*ferS*, which unexpectedly led to gains of functions in conidial germination and virulence against insects. Comparative transcriptomes between the wild type and Δ*ferS* suggest several potential genes associated with ferroptosis, oxidative stress response, ergosterol biosynthesis, TCA cycle, and mitochondrial expansion. These processes might serve as acquired oxidative stress responses, which promote oxidative stress resistance of Δ*ferS* during *B. bassiana* infection.

## Results and discussion

### The multimodular ferricrocin synthetase gene in *B. bassiana* BCC 2660

**Figure 1:**
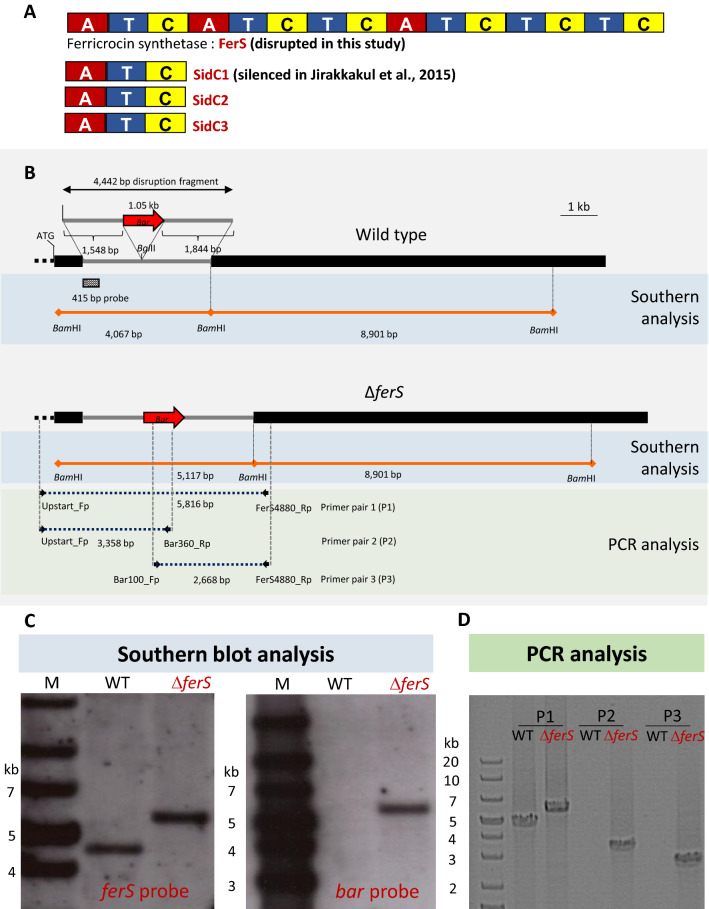
Targeted gene disruption of *ferS* using *Agrobaterium*-mediated transformation with the *bar* integration in *B. bassiana* BCC 2660. (**A**) The multimodular nonribosomal siderophore synthestase ‘FerS’ and three monomodular SidC-like proteins in the fungus. (**B**) Targeted disruption of *ferS* by the integration of the *bar* cassette at the *Bgl*II site of the *ferS* locus. For Southern analysis, the genomic DNA was restricted by *Bam*HI, and a 415-bp *ferS* fragment was used as a probe. Three primer pairs used in PCR analysis of the integration site and their locations relative to the *ferS* locus are indicated. (**C**) Southern analysis of Δ*ferS* and wild type hybridized by two DNA probes, *ferS* and *bar* fragments. (**D**) PCR analysis of Δ*ferS* and wild type using the three primer pairs. DNA standard sizes are shown on the left of each gel picture.

Before the complete genome of *B. bassiana* BCC 2660 was obtained and analyzed, the function of a *sidC*-like gene was determined by RNA silencing. The *sidC1*-silenced mutants showed deficiency in production of des-ferricrocin and ferricrocin, and had an increase in tenellin and iron-tenellin complex in iron-replete conditions^13^. However, the *B. bassiana* BCC 2660 genome sequence^14^ revealed that the fungus has four *sidC*-like genes, which are three monomodular NRPSs, *sidC1* (accession No. MZ086759; encoding a 1525-aa protein), *sidC2* (MZ086760; a 1417-aa protein) and *sidC3* (MZ086761; a 1380-aa protein), and a multimodular NRPS ‘*ferS*’ (MZ031022) that encodes a 4818-aa protein. The domain organization of each putative SidC-like protein is shown in Fig. 1A. All the three SidC-like NRPSs comprise only one set of A, T and C domains. By contrast, FerS consists of three complete modules of A-T-C, an extra set of T-C domains interrupted between the second and third modules, and a double set of the T-C domains at the C terminus. The monomodular SidC1 alone might not confer the ferricrocin biosynthesis based on its domain composition. Since there was a sequence similarity (33%) between s*idC1* and the first adenylation domain of *ferS*, the off-target effect of RNA silencing might account for the reduction in ferricrocin production in our previous study^13^. Therefore, in this study, the function of the putative ferricrocin synthetase gene *ferS* in *B. bassiana* BCC 2660 was verified by insertional mutagenesis.

**Figure 2:**
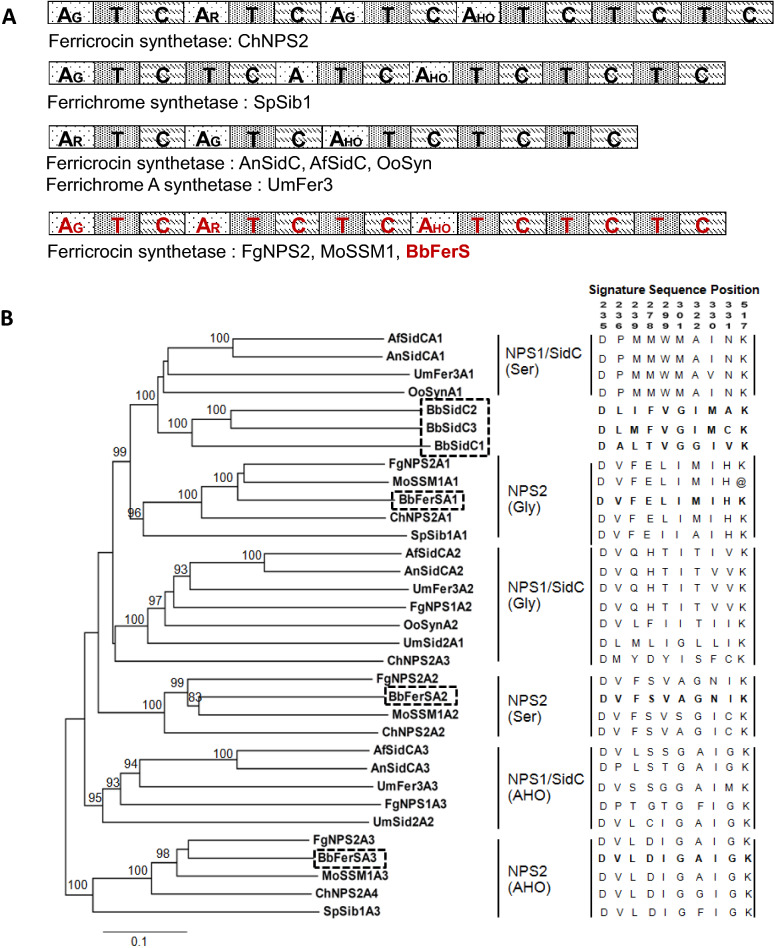
*Beauveria bassiana* BCC 2660 *ferS* and three SidC-like nonribosomal peptide synthetases (monomodular SidC1, SidC2 and SidC3) and sequence relationships with other ferricrocin and ferrichrome synthetases. (**A**) Domain organization of adenylation domain (A), thiolation domain (T), and condensation domain. The predicted amino acid substrate for each A domain is indicated. Abbreviations for these amino acids are as follow: HO, *N*^5^-acetyl-*N*^5^-hydroxyornithines; G, glycine; and Ser, serine. (**B**) Phylogenetic tree of the A domains of ferricrocin and ferrichrome synthetases was constructed using the neighbor-joining method. Bootstrap supports are percentages of 1000 replicates, and values of ≥ 80% are shown. *B. bassiana* A domains of FerS and three SidC-like NRPSs are highlighted in rectangles. The proteins used in this phylogenetic analysis are given in the Methods. Fungal ferrichrome synthetases are divided into two lineages, NPS1/SidC and NPS2. Accession numbers of all the NRPSs used in this phylogeny are provided in Supplemental File S5.

We have assessed the evolutionary conservation of *B. bassiana* BCC 2660 ferricrocin synthetase and their homologs. The domain architecture of FerS is remarkably similar to the modular architecture of ferrichrome synthetases (type IV NRPSs) such as NPS2 from *F. graminearum* and SSM1 from *M*. *grisea*^10^ (Fig. 2A). We performed multiple alignment of the adenylation domains from *B. bassiana* BCC 2660 FerS and the three monomodular SidCs and other known fungal ferrichrome and ferricrocin synthetases, and constructed a phylogenetic tree (Fig. 2B) using the neighbor-joining method in CLUSTAL-X^15^. The NRPS signature sequences for substrate specificity were also predicted by NRPS-PKS, which is a knowledge-based resource for analyzing nonribosomal peptide synthetases and polyketide synthases^16^. Amino acid residues at the signature sequences of adenylation domains from the four *B. bassiana* BCC 2660, including FerS, were compared to other known ferrichrome and ferricrocin synthetases (Fig. 2B). The phylogeny indicated that *B. bassiana* BCC 2660 FerS and three SidC-like NRPSs could be placed in two lineages, NPS1/SidC and NPS2, according to the previous classification^10^. The monomodular SidC-like NRPSs were clustered with the first adenylation domains of *A. nidulans* and A. *fumigatus* SidCs, which have substrate specificity to serine (Fig. 2A,B). Nevertheless, the signature sequences of the three monomodular SidCs do not match the signature sequence of the adenylation domains that are specific for serine, and neither do the signature sequences of adenylation domain in other ferrichrome and ferricrocin synthetases. On the other hand, FerS was clustered with ferricrocin synthetases in the NPS2 lineages. The signature sequences of all FerS adenylation domains were identical with the adenylation domains of *F. graminearum* ferricrocin synthetase NPS2 (FgNPS2); the first adenylation domain is specific for glycine, the second domain for serine, and the third domain for *N*^5^-acyl-*N*^5^ hydroxy-L-ornithines (AHO). Thus, our sequence analysis suggested that FerS is a complete ferricrocin synthetase, most likely crucial for ferricrocin biosynthesis in *B. bassiana* BCC 2660. The three SidC-like monomodular NRPSs could result from evolutionary events that include deletion of the second and third adenylation domains and a following triplication of the first adenylation domain.

### The *ferS*-null mutants abolished the ferricrocin production

Transformation of *B. bassiana* BCC 2660 with the *ferS*-disruption plasmid pCXFB4.4 generated 28 glufosinate-resistant transformants. Southern analysis indicated that two out of 28 transformants had an integration of the *bar* cassette at the targeted *ferS* locus, demonstrated by an increase of the 4-kb *fer*S fragment by the 1-kb size of *bar* (Fig. 1B). The Southern result also confirmed the presence of *bar* in the transformant but not in the wild type (Fig. 1B). Furthermore, our PCR analysis verified the similar *bar* integration in the same locus of *ferS* and the 5′ and 3′ border regions of the *bar* integration site (Fig. 1C).

**Figure 3:**
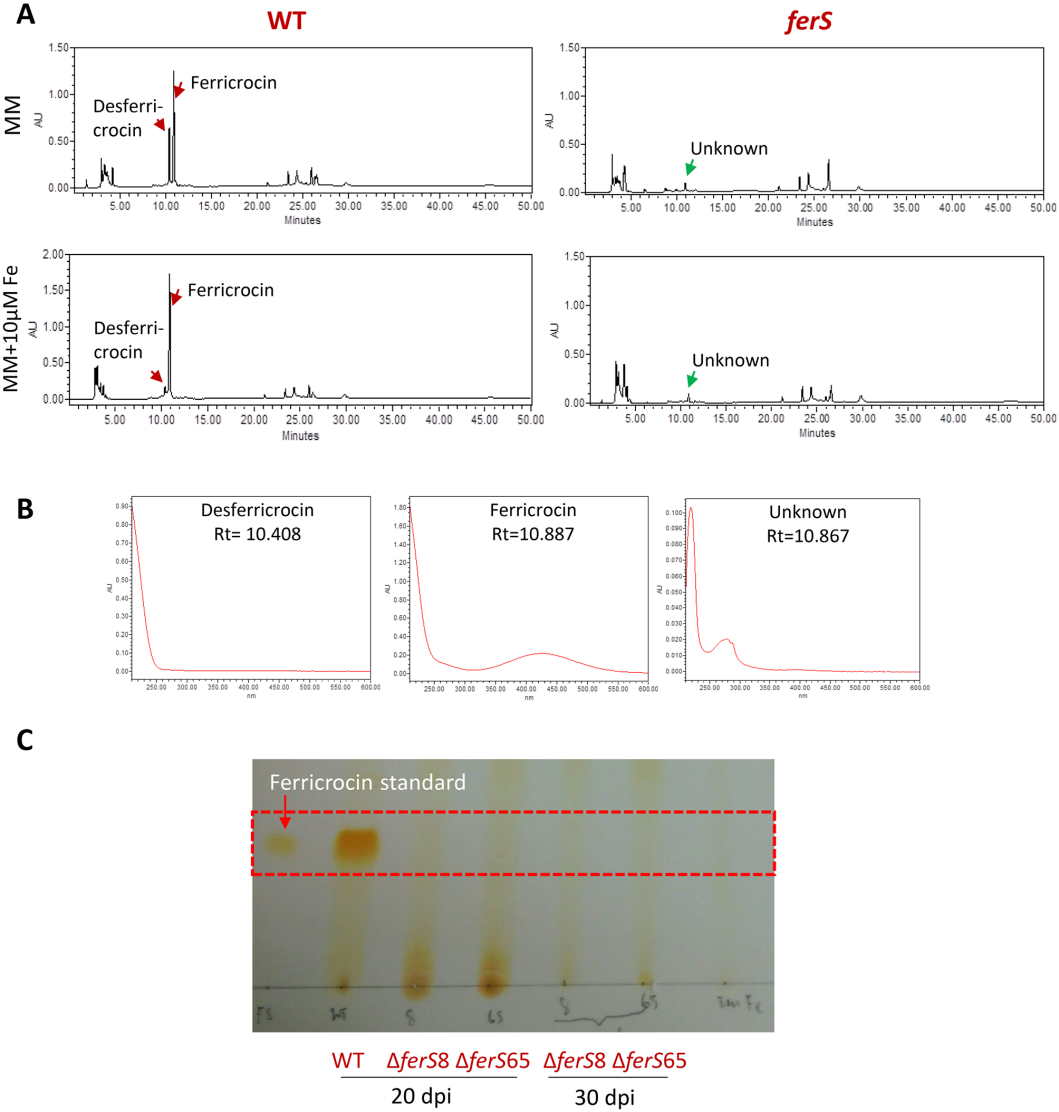
HPLC and TLC analysis of the mutant Δ*ferS* and wild type. (**A**) HPLC chromatogram of methanol extracts from *B. bassiana* cells of the wild type and Δ*ferS* under the iron-limited minimal medium (MM) and the iron-replete condition (MM containing 10 µM FeSO4). The peaks of ferricrocin, desferricrocin, and an unknown peak are indicated. (**B**) Spectrum absorption of ferricrocin, desferricrocin, and the unknown peak. Retention time (Rt) of these three peaks is provided. (**C**) TLC analysis of the cell extracts from two different strains of the two Δ*ferS* mutants, Δ*ferS*8 and Δ*ferS*65 and wild type on the 20^th^ and 30^th^ days of incubation. The ferricrocin was included as a reference.

Then, our metabolite analysis using HPLC indicated the lack of desferricrocin and ferricrocin production in Δ*ferS* (Fig. 3A). The metabolite profile of mycelial extracts from wild type showed desferricrocin and ferricrocin production at the retention time (Rt) of 10.408 and 10.887 min, respectively. Under the iron-replete conditions, the amount of ferricrocin has increased, while the amount of desferricrocin drastically decreased in the wild-type extract. The spectrum absorption of desferricrocin and ferricrocin are shown in Fig. 3B. In contrast, both the desferricrocin and ferricrocin peaks were undetected in the metabolite profile from Δ*ferS* (Fig. 3A). Notably, the Δ*ferS* metabolite profile had an unknown compound (c) peak at Rt of 10.867 min with the distinct spectrum absorption from those of ferricrocin and desferricrocin (Fig. 3B). We have analyzed the mycelial extracts of both wild type and Δ*ferS* using TLC, and verified that the mutant Δ*ferS* had abolished the ferricrocin production (Fig. 3C).

### The *ferS* disruption affected radial growth, germination and conidiation

**Figure 4:**
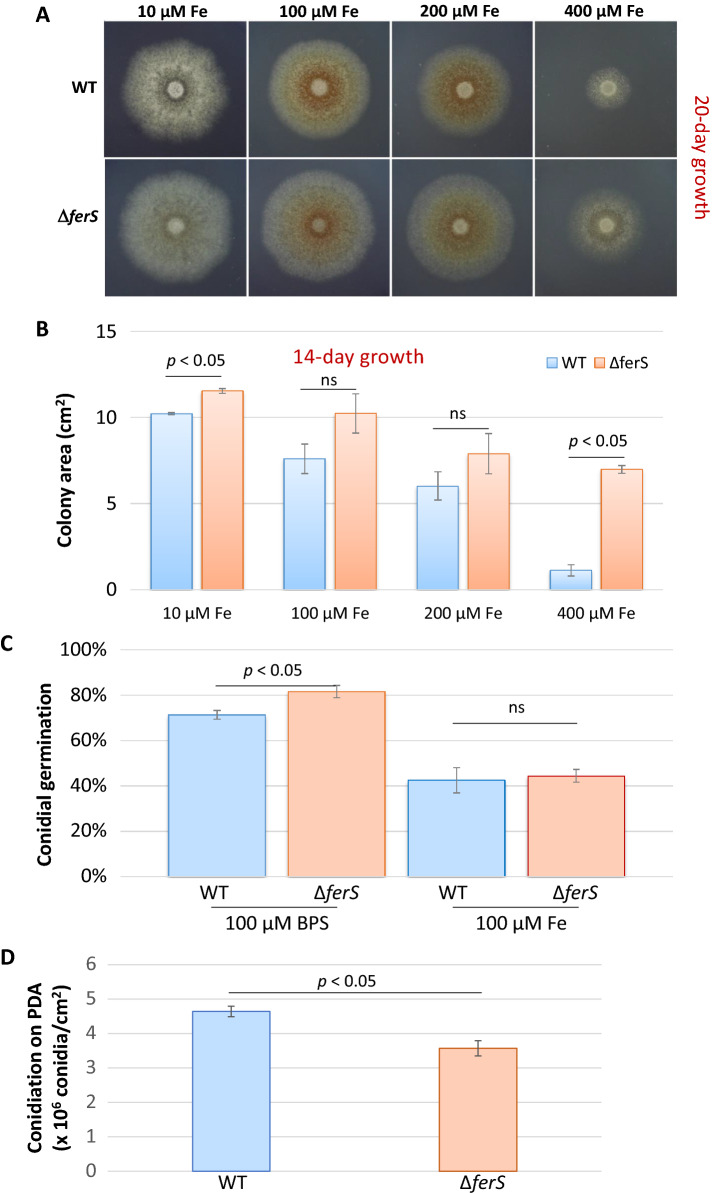
Effects of *ferS* disruption on the growth and developmental phenotypes of *B. bassiana*. (**A**) Colony appearances of Δ*ferS* and wild type (WT) on minimal medium containing 10, 100, 200 or 400 µM FeSO4 during a 20-day experimental period. (**B**) Radial growth of Δ*ferS* and wild type, determined by colony area, on a minimal medium containing 10–400 µM FeSO4 on the 14^th^ day of incubation. (**C**) Conidial germination under MM + BPS and MM + 100Fe. (**D**) Conidiation on potato dextrose agar on the 14^th^ day of incubation. Data shown are mean ± S.E.M. Statistical significance between wild type and Δ*ferS* (Student's *t* test: *p* < 0.05) is given on top of the bar graphs. ns, not significant.

The mutant Δ*ferS* surprisingly had some particular advantages in growth and development over the wild type. For the radial growth, as a mean of vegetative, hyphal growth, Δ*ferS* grew larger than the wild type on the same day of incubation under all the culture conditions supplemented by 10–400 µM Fe (Fig. 4A,B). At the low (10 µM) iron condition, the mutant radial growth increased by 13% over the wild type. When the iron concentrations were increased to 100 and 200 µM, the growth increases were more pronounced by 31–35% in Δ*ferS*. At the highest Fe concentration tested, the mutant grew bigger than the wild type by 400%, which was clearly observed by visual colony inspection (Fig. 4A,B). Under the iron depletion (MM + bathophenanthrolinedisulfonic acid (BPS); conducted in separate independent experiments), the mutant radial growth increased by 11% over the wild type. The *sidC1*-silenced mutants also increased radial growth compared to wild type under minimal medium agar supplemented by 10 µM Fe^13^. Conidial germination was also enhanced in Δ*ferS*. Our microscopic observation data indicated that Δ*ferS* conidia germinated at a significantly (*p* < 0.05) higher percentage than the wild-type conidia under the iron depletion (Fig. 4C), remarkably similar to the increase in the vegetative (hyphal) growth described above. However, under the iron-replete conditions, both the strains germinated similarly. Together, iron appears not necessary for the hyphal growth (shown by the data of radial growth and conidial germination) in *B. bassiana* BCC 2660, and indeed appears to have an inhibitory effect on vegetative growth.

In contrast, asexual reproduction, as a measurement of conidiation, was reduced in Δ*ferS*, consistent with a decreasing trend in conidiation found in *sidC1*-silenced mutants (Supplemental File S1). On potato dextrose agar (PDA) cultivation, the mutant produced a smaller number of conidia than the wild type (*p* < 0.05) per area of PDA culture (Fig. 4D). There was a clear distinction in aerial hyphae formation and conidiation between the wild type and ‘the ferricrocin-deficient/ferricrocin-free mutants’. The wild-type colony had a lawn of aerial mycelia and numerous, dense clusters of conidia; however, the mutants' colonies appeared to have sparse growth with fewer conidial clusters (Supplemental File S1). In *A. fumigatus*, ferricrocin is responsible for iron transport and distribution, especially iron transport from substrate hypha to the aerial parts. Therefore, the ferricrocin deficiency results in a reduction of conidial production^7^. Similarly, the reduction of both aerial hyphae and conidiation results suggested that the reduction or the abolishment in ferricrocin production impaired the development of aerial hyphae, conidiophores, and conidia in *B. bassiana* BCC 2660 mutants. Thus, the role of ferricrocin in the iron supply used for asexual development has been demonstrated in this study.

### The ferricrocin-free mutants had increased insect virulence

**Figure 5:**
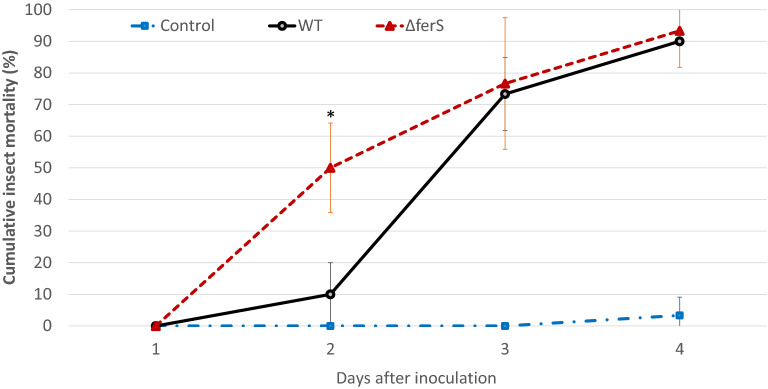
Virulence against beet armyworm (*Spodoptera exigua*) using intrahaemocoelic injection of the 30,000-conidium suspension of Δ*ferS* and wild type. Data shown are mean ± S.E.M. (from three independent experiments). Statistical significance between wild type and Δ*ferS* (Student's *t* test: *, *p* < 0.05) is given.

The mutant Δ*ferS* lacks ferricrocin, an important iron-storage molecule. As iron is essential for the pathogenicity of several pathogens in the hosts, the lack of ferricrocin in the mutant would have been assumed to lead to a deficiency in the virulence against the insect. However, our insect bioassay data from three independent experiments showed that Δ*ferS* was not deterred in the virulence against insect, compared to the wild type (Fig. 5). Indeed, the mutant was significantly increased in the ability to kill the insects, compared to wild type, on day 2 after inoculation (Fig. 5). The LT_50_ of Δ*ferS* was 2.46 days, 7 h shorter than wild type (LT_50_ of 2.75 days). This is interesting because we would not have anticipated a gain of function from a gene deletion unless the gene serves as a repressor or negatively relates to the phenotype.

### Comparative transcriptomes indicated differential gene expression patterns in response to iron depletion and iron excess between the mutant Δ*ferS* and wild type

We investigated what mechanisms that can lead to the increases in radial growth, germination, and insect virulence in Δ*ferS* as we observed. RNA Seq was conducted to compare the gene expression of wild type and Δ*ferS* under iron-depleted conditions (WT- and Δ*ferS*-BPS) and under iron-replete conditions (WT- and Δ*ferS*-Fe). These conditions were used to mimic the host–pathogen interaction process. The pathogen *B. bassiana* encounters the iron-limited environment at an early stage of infection, and the oxidative burst from the host defense response in the insect hemocoel. Our transcriptomic analysis with Cufflinks showed a total expression of 9879 genes and 10,066 isoforms in all eight replicates (each of the four treatments having two replicates). The pairwise comparison results identified 308 differentially-expressed genes (DEGs) (*p* < 0.01). Wild-type responses to iron-replete conditions were represented by the expression of 58 up-regulated DEGs and 41 down-regulated DEGs, of which 93 and 90% have putative known functions (Table 1). In Δ*ferS*, 41 DEGs were up-regulated, and 46 were down-regulated, of which 88 and 76% have putative functions under the iron-replete conditions (Table 1). The enriched functions of up-regulated DEGs in Δ*ferS* included cytochrome P450 and ABC transporter genes. In contrast, the enriched functions of down-regulated DEGs included those of coagulation factor, ricin b, and TauD.

**Table 1:** Comparative transcriptomic analysis between treatments [iron-depleted vs iron-replete conditions].

Pairwise comparison between treatments	Number of DEGs		Number of DEGs that has putative functions	
	Up-regulated	Down-regulated	Up-regulated	Down-regulated
WTBPS vs WTFe	58	41	54	37
FerBPS vs FerFe	41	46	36	35
WTBPS vs FerBPS	38	27	30	19
WTFe vs FerFe	23	34	19	25

Furthermore, the enriched DEGs were classified into 11 clusters based on gene expression patterns among four treatments using K-means clustering (k = 11) (Supplemental File S2). The overview of the expression profile of the clusters is shown in the graph. The bold black line is the medoid line that demonstrates the trend of expression profile in each DEG cluster. The complete list of clustering results is provided in Supplemental File S3. The expression profile of DEG clusters was evaluated in relation to gene functions and the pathway in which they involve. The KofamKOALA^17^ and BlastKOALA^18^ web tools were used to map DEGs into KEGG gene accession and pathways. There were 123 DEGs that can be mapped to the KEGG database. Among these DEGs, 46 genes were specific for, i.e., have the highest specificity scores in, the iron-depleted conditions (30 genes in WT-BPS and 16 genes in Δ*ferS*-BPS). By contrast, 77 genes were specific for the iron-replete conditions (19 genes in WT-Fe and 58 genes in Δ*ferS*-Fe).

DEG clusters 2 and 5 included genes that had increased expression in Δ*ferS* over wild type under iron-depleted and replete conditions (Supplemental File S2). In cluster 2, the long-chain acyl-CoA synthetase gene *acsl4* is involved in ferroptosis, a regulated form of cell death and characterized by a production of reactive oxygen species (ROS) from accumulated iron and lipid peroxidation. ACSL4 regulated ROS via phospholipid-hydroperoxide glutathione peroxidase. We also found the low-affinity ferrous iron transporter gene *fet4* in cluster 2. Cluster 5 contained six DEGs, four of which have not been characterized for functions in the literature. It is fascinating that under the iron-depleted condition, cluster 5′s DEGs were not expressed at all in wild type but strongly expressed in Δ*ferS*. These could be the genes that were apparently induced in Δ*ferS* in response to iron depletion. Understanding the functions of these genes would lead to finding de novo proteins that have essential roles in response to no or low iron conditions in fungi.

On the other hand, cluster 11′s DEGs had the opposite expression pattern to those in clusters 2 and 5. These genes' expression was utterly missing in Δ*ferS*, but was high in the wild type under the iron-replete conditions. One of these genes was the ferric reductase required for the high-affinity iron uptake^19^, suggesting that Δ*ferS* might be impaired in the reductive iron uptake. A likely hypothesis for this phenomenon might be to limit or reduce the level of labile Fe^2+^ in the Δ*ferS* cells, which often causes iron toxicity. Moreover, as reported above Δ*ferS* exhibited the increased virulence against the insect host. This is strikingly similar to the hypervirulence phenotype found in the mutant Δ*fet1* knocked-out in the ferroxidase gene, a core component of the reductive iron assimilation system in the phytopathogen *Botrytis cinera*^20^.

**Figure 6:**
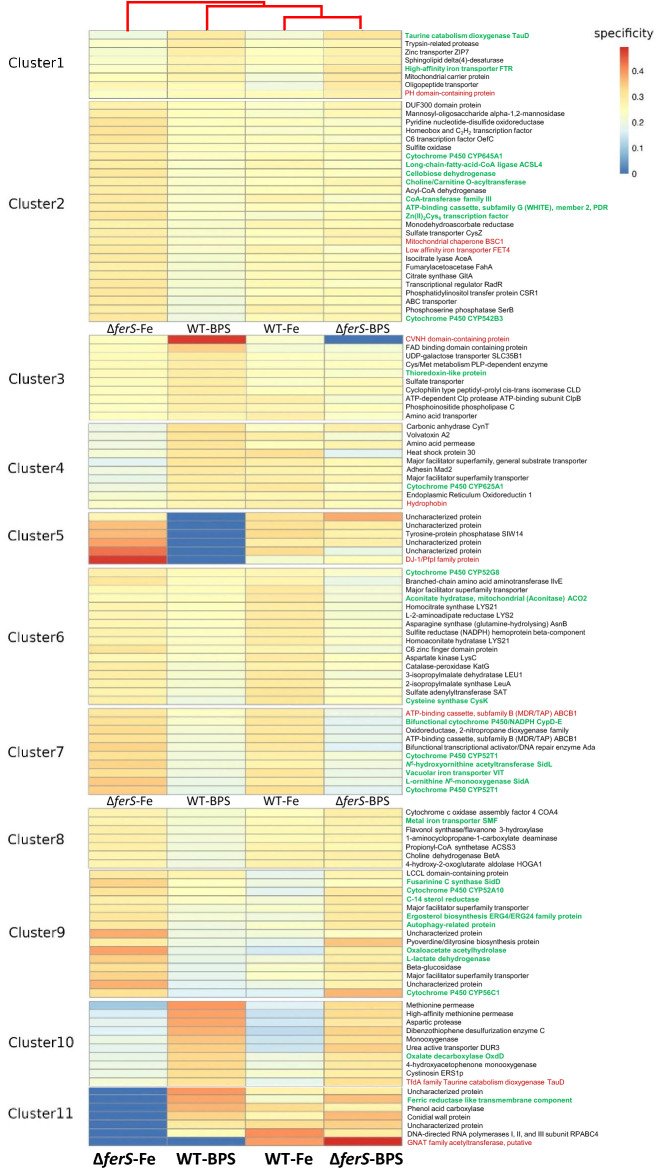
Expression levels of DEGs based on the specificity scores in four treatment conditions: WT-BPS, Δ*ferS*-BPS, WT-Fe, and Δ*ferS*-Fe. DEGs were also classified in 11 clusters with distinct expression patterns.

Cluster 9 was particularly intriguing that the mutant Δ*fer*S was considerably increased in expression of fusarinine C synthase, cytochrome P450 52A10, cytochrome P450 CYP56C1, C-14 sterol reductase, ergosterol biosynthesis ERG4/ERG24 family protein, autophagy-related protein, oxaloacetate acetylhydrolase, L-lactate dehydrogenase and two major facilitator superfamily transporters, compared with wild type (Fig. 6). The data of the other clusters are provided in Fig. 6 and Supplemental Files. S2 and S3.

### Increase in certain parts of siderophore biosynthesis and other iron homeostasis mechanisms in Δ*ferS*

The wild type and Δ*ferS* had a notably similar pattern of gene expression in three siderophore biosynthetic genes, *sidA, sidD,* and *sidL,* under the iron-depleted condition. On the other hand, when the fungal cells were exposed to the high-iron condition, *sidA*, *sidD,* and *sidL* were markedly enhanced in the expression in the mutant Δ*ferS* (Fig. 6). SidD is a nonribosomal siderophore synthetase required for biosynthesis of the extracellular siderophore, fusarinine C. Its production is usually induced upon a low-iron environment, and suppressed upon a high-iron condition^21^. Here, in Δ*ferS* the expression of *sidD* was significantly more pronounced than in wild type in the iron-replete conditions (Fig. 6). The wild-type *sidD* expression level under the iron-replete conditions decreased to half of that in iron depletion. However, there was no clear statistical significance in the difference (*p* = 0.058). On the other hand, Δ*ferS* had significantly enhanced expression of *sidD* to a threefold under the iron-replete conditions, compared to that under iron depletion (*p* = 0.006). The siderophore-mediated iron homeostasis regulation could sense the unavailability of ferricrocin and, in turn, produce and secrete fusarinine C for sequestering iron. Such a higher level of fusarinine C could promote the infection of Δ*ferS* in the host, as we observed the higher insect virulence of the mutant than the wild type. Inside the cell, SidL is *N*^5^-hydroxyornithine-acetylase required for biosynthesis *N*^5^-acetyl-*N*^5^-hydroxyornithine, an essential intermediate of ferricrocin biosynthesis. The expression of *sidL* was drastically increased to 26.9-fold in Δ*ferS* (*p* < 5E^−05^), but to only 5.0-fold in the wild type (*p* < 5E^−05^) when the expression in iron-replete conditions was compared to that in iron deplete (Fig. 6). The drastic increase of *sidL* expression could be due to the similar regulatory mechanism that senses no ferricrocin in the cell. Lastly, SidA is L-ornithine *N*^5^-monooxygenase essential for biosynthesis of *N*^5^-hydroxy-L-ornithine, the building block of all siderophores in fungi. Similarly to the *sidL* expression pattern with a less extent, the expression of *sidA* was increased to 5.2-fold in Δ*ferS* (*p* < 5E^−05^), but to only 3.4-fold in the wild type (*p* < 5E^−05^) when expression in iron-replete conditions was compared to that in iron depletion (Fig. 6).

In addition to those in siderophore biosynthesis, the iron homeostasis genes had differential gene expression patterns under the iron-replete conditions. The vacuolar iron transporter (*vit*) gene was up-regulated in response to the high iron condition by an increase of 58.5-fold in Δ*ferS* (*p* < 5E^−05^), but 31.3-fold in the wild type (*p* < 5E^−05^). In contrast, reductive iron assimilation-related genes such as iron transport multicopper oxidase (*fet3*) and high-affinity iron transporter (*ftr*) genes were down-regulated under high iron conditions. Nevertheless, for *fet3*, the mutant Δ*ferS* had a two-fold expression level over that of wild type under low and high iron conditions (Fig. 6).

### Δ*ferS* was increased in ferroptosis, oxidative stress response, ergosterol biosynthesis, TCA cycle, and mitochondrial expansion

Interestingly, Δ*ferS* showed remarkable up-regulation of genes for cytochrome P450 and those in TCA cycle, ergosterol biosynthesis, alternative iron homeostasis, autophagy, and ferroptosis under iron depletion iron-replete conditions, compared to the wild type.

### Ferroptosis, oxidative stress response and ergosterol biosynthesis

The oxaloacetate acetylhydrolase and cellobiose dehydrogenase (CDH) genes were up-regulated in Δ*ferS,* especially in the high iron environment. Oxaloacetate acetylhydrolase is involved in oxalate production. The gene was up-regulated in Δ*ferS,* especially in iron-replete conditions. In the meantime, oxalate decarboxylase gene, required for decomposition of oxalate to formate and carbon dioxide^22^, was down-regulated in Δ*ferS*. Oxalate can reduce the toxicity of metals by forming metal-oxalate complexes, therefore being able to act as an iron chelator. The formation of iron oxalates has been reported in *B. bassiana*^23^. The CDH is a heme-containing oxidoreductase that can transfer electrons to electron acceptors such as cytochrome c and ferric-oxalate^24^. CDH has an essential role in wood decomposition^25,26^. This oxidoreductase can generate hydrogen peroxide by oxygen reduction and helps degrade cellulose, xylan, and lignin in the presence of hydrogen peroxide and ferrous ions^24,27^. Therefore, the up-regulation of oxaloacetate acetylhydrolase and CDH in Δ*ferS* is consistent with the process that leads to the ferroptosis pathway via the Fenton reaction and lipid peroxidation. Oxalate binds to Fe^3+^ to form iron-oxalate complex. CDH acts as a hydrogen peroxide (H_2_O_2_) generator and iron-reducing agent, which reduces Fe (III)-oxalate complex to ferrous ions (Fe^2+^). The accumulation of Fe^2+^ in the cytoplasm induced the expression of vacuolar iron transporter (VIT). The mutant Δ*ferS* had a significant (*p* < 5E^−05^) increase of *vit* expression compared to wild type (Fig. 6). The coincidence of Fe^2+^ and H_2_O_2_ could lead to hydroxyl radical generation via the Fenton reaction.

The generation of such free radicals can damage the cell membrane by the process of membrane lipid peroxidation. However, our transcriptomic data indicated that ergosterol biosynthesis genes and oxidative stress response gene were up-regulated in Δ*ferS*, compared with wild type (Fig. 6). These ergosterol biosynthesis genes included genes for ergosterol biosynthesis proteins ERG4/ERG24 and C-14 sterol reductase. The oxidative stress response genes included catalase peroxidase (katG), glutathione transporter, autophagy-related protein (ATG22), and Zn(II)2Cys6 type transcription factor. Catalase peroxidase is an antioxidant enzyme that is active in response to H_2_O_2_ accumulation in fungal cell^28^. ATG22 is a vacuolar efflux of amino acids, which helps maintain protein synthesis and viability under nitrogen starvation during the autophagy-associated processes^29^. Nitrogen starvation is related to oxidative stress and membrane peroxidation^30^. Interestingly, the ATG22 homolog of *B. bassiana* has been reported to be involved in fungal pathogenicity^31,32^.

*Bbpc1* and *BbThm1* encode Zn(II)2Cys6 type transcription factors in *B. bassiana*. Bbpc1 plays a role in oxidative stress response, virulence, and conidial and blastospore production^33^. BbThm1 has been reported as a regulator of membrane homeostasis and heat and sodium/lithium dodecyl sulfate (S/LDS) stress^34^. In *A*. *fumigatus*, Zn(II)2Cys6 type transcription factor AtrR has been reported to be involved in ergosterol biosynthesis, adaptation in hypoxia condition, and virulence. The cytochrome P450 14-alpha sterol demethylase, Cyp51A is an iron-dependent enzyme and a target of Zn2-Cys6 Transcription Factor (AtrR) in ergosterol biosynthesis^35^. Ergosterol can protect lipid against peroxidation, and the increasing ergosterol level in the cell membrane can inhibit the membrane damage and sustain membrane permeability^36,37^.

Furthermore, a positive correlation between ergosterol biosynthesis and the ability of oxidative stress protection has been demonstrated in *Saccharomyces cerevisiae*^38^. Therefore, the notably increased expression of stress response genes and ergosterol biosynthesis genes in Δ*ferS* in both iron-replete and iron-depleted conditions might result from the cell acclimation processes. This cell acclimation occurred during oxidative stress conditions, generated from the Fenton reaction in the iron excess and oxidative stress induced by iron starvation. In iron starvation, some iron-dependent mechanisms such as oxidative phosphorylation can be affected and lead to ROS generation^39^.

### TCA cycle and mitochondrial expansion

**Figure 7:**
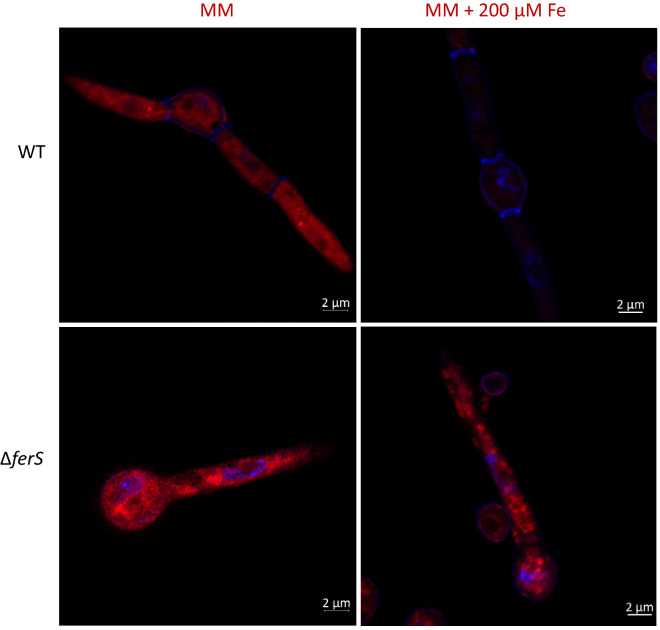
Mitochondrial observation in Δ*ferS* and wild type on minimal medium (MM) and MM containing 200 µM FeSO4 (MM + 200Fe) during a 16-h incubation. Fungal cells were stained with MitoTracker Deep Red, counter-stained with DAPI, and visualized using confocal laser scanning microscopy. Bars, 2 µm.

In the viewpoint of primary metabolism, under iron-replete and iron-depleted conditions, Δ*ferS* showed higher expression levels of genes involved in TCA cycle and the central carbon metabolism such as citrate synthase (gltA), L-lactate dehydrogenase (ldh) isocitrate lyase (Icl1), and choline/carnitine O-acyltransferase, compared to the wild type (Fig. 6). These results might be consequences of mitochondrial expansion and increased iron pool in mitochondria, promoting TCA cycle activity. In this study, the expansion of mitochondria in Δ*ferS* was clearly detected using fluorescence staining, compared to the wild type. The mitochondrial expansion was found under both iron-depleted and replete conditions, suggesting a constitutive pattern (Fig. 7). In contrast, wild-type mitochondria were expanded only under iron depletion (Fig. 7). The wild-type occurrence was consistent with the phenomenon in *Saccharomyces cerevisiae*, in which the yeast cells can expand the mitochondrial compartments during iron starvation due to diauxic shift condition^40^. On the other hand, the Δ*ferS* mitochondrial expansion occurred regardless of iron availability. The expansion in mitochondrial volume leads to an increase of iron pool in mitochondria, which induces the expression of high-affinity iron transporter such as Fet3 and Ftr1 under iron starvation, as reported in *S. cerevisiae*^41^. The expansion of the mitochondrial compartment, as well as mitochondrial iron pool, was consistent with the increase in heme and Fe-S cluster-dependent proteins in TCA cycle and respiratory complexes in Ascomycetes^40^.

In conclusion, Δ*ferS* that lacks intracellular siderophore ferricrocin responds to iron-depleted and iron-replete conditions using specific processes. Both iron starvation and iron excess can result in ROS generation. The ferricrocin-free mutant produced oxalate (predicted by transcriptomic data) as an iron chelator. However, the induced expression of CDH could generate H_2_O_2_ and promote ROS production (via the Fenton reaction), lipid peroxidation, and ferroptosis. Therefore, the mutant Δ*ferS* might sense the iron excess and the oxidative stress. In turn, the antioxidant-related genes, ergosterol biosynthesis and TCA cycle was up-regulated under both iron-depleted, and iron-replete condition. These responses are potentially analogous to the priming, in which the Δ*ferS* cells are trained for adaptation to severe stresses. Therefore, these increased biological pathways empower the mutant Δ*ferS* during the host infection and lead to higher insect mortality than the wild type in the early phase of infection.

## Methods

### Fungal strain and culture conditions

*Beauveria bassiana* BCC 2660 was a biological control strain from the Thailand Bioresource Research Center in Thailand. The wild type and transformants were maintained on potato dextrose agar (PDA; Difco, USA) or PDA containing 100 μg mL^−1^ of glufosinate ammonium (Zhejiang Yongnong Chem, China), respectively, at 25–28 °C. For insect bioassay, a conidial suspension was harvested from a 7-day-old PDA culture by resuspending the conidia in distilled water and filtering them through a sterile cheesecloth to remove mycelia. For assays under iron-depleted and iron-replete conditions, 1 × 10^7^ conidia mL^−1^ of the wild type or transformants were inoculated on minimal medium [MM; 1% dextrose (Difco) and 20 mM glutamine (Sigma-Aldrich, USA)^8^ containing 100 μM bathophenanthrolinedisulfonic acid (Sigma-Aldrich) (MM + BPS) for the iron-depleted condition and MM containing 100–400 μM FeSO_4_ (Sigma-Aldrich) (MM + Fe) for the iron-replete condition.

*Escherichia coli* strain DH5α was used for bacterial transformation and recombinant plasmid propagation.

### Targeted disruption of the ferricrocin synthetase gene in *B*. *bassiana*

Targeted disruption of *B. bassiana* BCC 2660 *ferS* was performed by inserting a bialophos resistance (*bar*) cassette between the thiolation (T) domain and the condensation (C) domain in the first module of *ferS*. A 3392-bp *ferS* fragment was amplified from *B. bassiana* BCC 2660 genomic DNA with the primer pair FerS-F and FerS-R (Supplemental File S4). The *Xba*I restriction sites are included in the two primers for facilitating the cloning. The *ferS* fragment was cloned into the vector pCAMBIA1300 at the *Xba*I site to generate plasmid pCXF3.4. Next, the *bar* cassette was amplified from the plasmid pCB1534 using the primers Bar-F and Bar-R (Supplemental File S4). The underlined bases indicate the *Bgl*II restriction site. The pCXF3.4 was digested with *Bgl*II and then ligated with the *Bgl*II-restricted *bar* cassette. Therefore, we obtained the ferS-disruption plasmid pCXFB4.4, of which *ferS* is interrupted by the *bar* cassette (Fig. 1). The disruption vector pCXFB4.4 was transformed into *Agrobacterium tumefaciens* strain EHA 105 using the protocol described previously^42^ with some critical modifications^43^.

To determine the integration of the *bar* cassette in *ferS* transformants, the genomic DNA was analyzed by Southern and PCR analyses in glufosinate-resistant transformants, compared with the wild type. For Southern analysis, 10 ug of completely *Bam*HI-digested genomic DNA from wild type and *ferS* transformants were loaded onto 1% agarose gel electrophoresis, and the DNA was transferred and cross-linked to a nylon membrane (Hybond N + ; GE healthcare Bio-sciences, U.S.A.). The 415 bp of *ferS* fragment was non-radioactively labeled using an alkaline phosphatase-based system (CDP-Star; GE Healthcare Bio-Sciences). The hybridization was performed with the CDP-Star-labelled *ferS* fragment probe at 55 °C overnight. After high stringency wash, the membrane was incubated with CDP-Star detection solution and exposed to X-ray film (Hyperfilm_ECL; GE Healthcare Bio-Sciences). PCR analysis was performed by three primer pairs. The first pair was used to amplify a *ferS* region covering the *bar* integration site and includes Upstart_Fp and FerS4880_Rp (Supplemental File S4). The second and third primer pairs were used to amplify the border regions between the *bar* cassette and the *ferS* locus at the *bar*'s 5′ and 3′ ends, respectively. The second pair included Upstart_Fp and Bar-360R. The third pair had Bar-100F and FerS4880_Rp (Supplemental File S4).

### HPLC and TLC analysis

Determination of ferricocin in wild type and Δ*ferS* were performed by HPLC analysis, as previously described^13^ with some modifications. *B. bassiana wild*-type or Δ*ferS* was grown on a cellophane sheet laid on top of MM or MM + 10 µM FeSO_4_. The culture was incubated at 25 °C for 20 days. The harvested mycelia were air-dried and extracted with 50 ml of methanol for 2 days. After discarding the mycelia, the methanol fraction was concentrated under reduced pressure to obtain a crude extract. HPLC analysis was conducted using a reverse-phase column (VertiSep HPLC Column; Vertical Chromatography, Thailand) and diode array detector (996 Photodiode Array Detector, Waters). The crude extract was dissolved in methanol to a final concentration of 10 mg ml^−1^. Metabolite separation was performed on a VertiSep HPLC Column. Analysis was performed at a flow rate of 0.8 ml min − 1 at 210 nm with a water–acetonitrile step gradient as follows: 0 min/2% acetonitrile, 14 min/60% acetonitrile, 16 min/60% acetonitrile, 19 min/100% acetonitrile, 50 min/100% acetonitrile, 51 min/50% acetonitrile, 60 min/50% acetonitrile and 64 min/2% acetonitrile.

For TLC analysis, the crude mycelial extracts were spotted on a TLC plate (TLC silica gel 60 F254 25 aluminum sheets 20 × 20 cm, Merck, Germany), and developed by a freshly prepared solvent chloroform/methanol/water (70:24:4) system, as previously reported^44^.

### Insect bioassay

We have compared the virulence against insects of *B. bassiana* wild type and Δ*ferS* using beet armyworm (*Spodoptera exigua*). We performed intrahaemocoelic injection of beet armyworms by using 3 µl of conidial suspension at the density of 1 × 10^7^ conidia mL^−1^ as previously described^14^. Control larvae were injected with saline (0.85% NaCl). The inoculated insect larvae were then placed and fed with the armyworm medium^14^ in a plastic container, kept in a large carton at 25 °C. The relative humidity inside the carton was maintained above 80% by using a fine-nozzle spray. There were ten beet armyworm larvae for each treatment, and the experiment was repeated four times. Insect mortality was determined at 24, 48, 72, 96, and 120 h post-inoculation (PI).

### Comparative analysis of radial growth, conidiation and conidial germination between Δ*ferS* and wild type

For radial growth determination, ferricrocin-deficient mutant *ΔferS* and the wild type were grown under the iron-depleted and iron-replete conditions, 10 μl of 1 × 10^5^ conidia mL^−1^ were inoculated at the center of MM, MM + BPS, MM + 100Fe and MM + 200Fe. The colony diameter was measured at 3, 5, 7, 9, and 12 days after inoculation. To determine conidiation, the number of conidia produced in a 1 × 1 cm^2^ area of culture was determined by using a hemocytometer 14 days after inoculation.

We conducted the germination assay in slide culture. For each strain, conidia were incubated in 200 µL of 5% PDB (v/v) containing 100 µM BPS (PDB + BPS) or 100 µM FeSO_4_ (PDB + 100Fe) broth for a final concentration of 1 × 10^6^ conidia mL^−1^ at 25 °C for 16 h. Conidial germination was determined by counting the number of germinated conidia relative to the total number of conidia in a hemocytometer. There were three replicates for each treatment, and the experiment was repeated three times.

### Comparative transcriptomic analysis under iron-depleted and iron-replete conditions

The wild type and Δ*ferS* strains of *B. bassiana* were cultured in MM + BPS and MM + 200Fe as described above for 4 h. The mycelia were harvested by filtration through cheesecloth and ground to the fine powder in liquid nitrogen, and total RNA was extracted using Ambion^TM^ TRIzol Reagent (Invitrogen, USA). For the four treatments (WT-BPS, WT-Fe, Δ*ferS*-BPS, and Δ*ferS*-Fe), there were two replicates (two sets of total RNAs) for each treatment. Total RNA quality and quantity were measured by NanoDrop One Microvolume UV–Vis spectrophotometer. Poly (A) mRNA was isolated from 75 µg of total RNA using Dynabeads™ mRNA Purification Kit (Thermo Fisher Scientific). Fragment Analyzer (Advanced Analytical Technologues) was used to quantify the concentration and quality of isolated mRNA by DNF-472M33 kit (HS mRNA 15nt). The mRNAs were used to construct RNA libraries using Ion Total RNA-Seq kit v2 protocol (Life Technologies). cDNA was synthesized using SuperScript® III Enzyme Mix, purified by magnetic bead cleanup module, and eluted in 6 µl of pre-heated nuclease-free water. Sequencing adapters and barcode adapters were ligated and amplified using Platinum® PCR SuperMix High Fidelity, Ion Express™ RNA 3′ Barcode primer, and Ion Express™ RNA-Seq Barcode BC primer. RNA libraries were sequenced using on 540™ Kit-OT2 on Ion S5™ XL.

The transcriptomic read data were mapped to the annotated genome of *B. bassiana* BCC 2660 using Cufflinks version 2.2.1^45^. The genome annotation was conducted using the MAKER annotation pipeline version 2.31.10^46^. The transcriptomic expression profile of each replicate was quantified into Fragments Per Kilobase Million (FPKM). The FPKM values were log-transformed and normalized using geometric normalization. The normalized data were imported to R version 4.0 and analyzed using cummeRbund package version 2.30.0^47^. The pairwise comparison was employed to determine the significant differentially expressed genes (DEGs) for each pair of experiment conditions (*p* < 0.01). In order to assess to which condition each DEG was specific, the specificity scores of DEGs in 4 treatment conditions (WT-BPS, Δ*ferS*-BPS, WT-Fe, and Δ*ferS*-Fe) were calculated using *csSpecificity* method in cummeRbund package. For functional assessment, the DEGs between wild type and Δ*ferS* in different conditions were classified into up-regulated and down-regulated groups. The functional enrichment analysis was then conducted using STRING v11 with a false discovery rate < 0.05^48^.

### Mitochondrial staining and confocal laser scanning microscopy

We have determined the distribution pattern of mitochondria in the fungal cells using MitoTracker staining and 4′,6-diamidino-2-phenylindole (DAPI) counter-staining. Germinating conidia were chosen for this staining, as the cells would undergo a high level of mitochondrial activity for conidial germination. *B. bassiana* wild type or the mutant Δ*ferS* was inoculated at the density of 1 × 10^6^ conidia/ml in iron-low (10%, v/v) PDB in sterile water or iron-replete (10% PDB containing 200 µM FeSO_4_) condition. The addition of the diluted PDB, instead of MM, speeds up the germination of conidia. Two hundred µl of conidial suspension was dropped on a glass slide and incubated inside a moisturized container at 25–28 °C for 16–18 h. The germinating conidia were then washed by phosphate buffer saline (PBS), pH 7.4. Conidia were fixed in 1 ml of 4% paraformaldehyde for 10 min at 25–28 °C, followed by washing twice with PBS. For staining, the conidia were stained with 1 ml of 250 nM MitoTracker Deep Red (Invitrogen) in the dark at 37 °C. After 60 min, 500 µl of the dye was removed from the sample, replaced by 500 µl of 0.25 µM DAPI and incubated 37 °C in the dark for 20 min. Slide cultures were then washed twice in PBS. The mitochondrial distribution in the cell was documented using confocal laser scanning microscope model LSM800 with Airyscan (Zeiss, Germany), as previously described^49^.

## Supplementary Information

Supplementary Information 1.

Supplementary Information 2.

Supplementary Information 3.

Supplementary Information 4.

Supplementary Information 5.

## References

[1] Crichton RR, Wilmet S, Legssyer R, Ward RJ (2002). Molecular and cellular mechanisms of iron homeostasis and toxicity in mammalian cells. J. Inorg. Biochem..

[2] Haas H, Eisendle M, Turgeon BG (2008). Siderophores in fungal physiology and virulence. Annu. Rev. Phytopathol..

[3] Renshaw JC, Robson GD, Trinci AP, Wiebe MG, Livens FR, Collison D, Taylor RJ (2002). Fungal siderophores: structures, functions and applications. Mycol. Res..

[4] Haas H (2003). Molecular genetics of fungal siderophore biosynthesis and uptake: the role of siderophores in iron uptake and storage. Appl. Microbiol. Biotechnol..

[5] Garrick MD (2011). Human iron transporters. Genes. Nutr..

[6] Peng YJ, Wang JJ, Lin HY, Ding JL, Feng MG, Ying SH (2020). HapX, an indispensable bZIP transcription factor for iron acquisition, regulates infection initiation by orchestrating conidial oleic acid homeostasis and cytomembrane functionality in mycopathogen *Beauveria bassiana*. mSystems..

[7] Wallner A, Blatzer M, Schrettl M, Sarg B, Lindner H, Haas H (2009). Ferricrocin, a siderophore involved in intra- and transcellular iron distribution in *Aspergillus fumigates*. Appl. Environ. Microbiol..

[8] Eisendle M, Schrettl M, Kragl C, Muller D, Illmer P, Haas H (2006). The intracellular siderophore ferricrocin is involved in iron storage, oxidative-stress resistance, germination, and sexual development in *Aspergillus nidulans*. Eukaryot. Cell..

[9] Gibson DM, Donzelli BG, Krasnoff SB, Keyhani NO (2014). Discovering the secondary metabolite potential encoded within entomopathogenic fungi. Nat. Prod. Rep..

[10] Bushley KE, Ripoll DR, Turgeon BG (2008). Module evolution and substrate specificity of fungal nonribosomal peptide synthetases involved in siderophore biosynthesis. BMC Evol. Biol..

[11] Hof C, Eisfeld K, Welzel K, Antelo L, Foster AJ, Anke H (2007). Ferricrocin synthesis in *Magnaporthe grisea* and its role in pathogenicity in rice. Mol. Plant. Pathol..

[12] Mei B, Budde AD, Leong SA (1993). sid1, a gene initiating siderophore biosynthesis in *Ustilago maydis*: molecular characterization, regulation by iron, and role in phytopathogenicity. Proc. Natl. Acad. Sci. USA.

[13] Jirakkakul J, Cheevadhanarak S, Punya J, Chutrakul C, Senachak J, Buajarern T, Tanticharoen M, Amnuaykanjanasin A (2015). Tenellin acts as an iron chelator to prevent iron-generated reactive oxygen species toxicity in the entomopathogenic fungus *Beauveria bassiana*. FEMS Microbiol. Lett..

[14] Toopaang W, Phonghanpot S, Punya J, Panyasiri C, Klamchao K, Wasuwan R, Srisuksam C, Sangsrakru D, Sonthirod C, Tangphatsornruang S, Tanticharoen M, Amnuaykanjanasin A (2017). Targeted disruption of the polyketide synthase gene pks15 affects virulence against insects and phagocytic survival in the fungus *Beauveria bassiana*. Fungal Biol..

[15] Thompson JD, Gibson TJ, Plewniak F, Jeanmougin F, Higgins DG (1997). The CLUSTAL_X windows interface: flexible strategies for multiple sequence alignment aided by quality analysis tools. Nucleic. Acids. Res..

[16] Ansari MZ, Yadav G, Gokhale RS, Mohanty D (2004). NRPS-PKS: a knowledge-based resource for analysis of NRPS/PKS megasynthases. Nucleic. Acids. Res..

[17] Aramaki T, Blanc-Mathieu R, Endo H, Ohkubo K, Kanehisa M, Goto S, Ogata H (2020). KofamKOALA: KEGG ortholog assignment based on profile HMM and adaptive score threshold. Bioinformatics.

[18] Kanehisa M, Sato Y, Morishima K (2016). BlastKOALA and GhostKOALA: KEGG tools for functional characterization of genome and metagenome sequences. J. Mol. Biol..

[19] Schröder I, Johnson E, de Vries S (2003). Microbial ferric iron reductases. FEMS Microbiol. Rev..

[20] Vasquez-Montaño E, Hoppe G, Vega A, Olivares-Yañez C, Canessa P (2020). Defects in the ferroxidase that participates in the reductive iron assimilation system results in hypervirulence in *Botrytis cinerea*. MBio.

[21] Misslinger M, Hortschansky P, Brakhage AA, Haas H (2020). Fungal iron homeostasis with a focus on *Aspergillus fumigatus*. Biochim. Biophys. Acta, Mol. Cell Res..

[22] Mäkelä MR, Hildén K, Lundell TK (2010). Oxalate decarboxylase: biotechnological update and prevalence of the enzyme in filamentous fungi. Appl. Microbiol. Biotechnol..

[23] Joseph E, Cario S, Simon A, Wörle M, Mazzeo R, Junier P, Job D (2012). Protection of metal artifacts with the formation of metal-oxalates complexes by *Beauveria bassiana*. Front. Microbiol..

[24] Zamocky M, Ludwig R, Peterbauer C, Hallberg B, Divne C, Nicholls P, Haltrich D (2006). Cellobiose dehydrogenase-a flavocytochrome from wood-degrading, phytopathogenic and saprotropic fungi. Curr. Protein Pept. Sci..

[25] Prasetyo EN, Rodríguez R, Lukesch B, Weiss S, Murkovic M, Katsoyannos E, Sygmund C, Ludwig R, Nyanhongo G, Guebitz G (2015). Laccase–cellobiose dehydrogenase-catalyzed detoxification of phenolic-rich olive processing residues. Int. J. Environ. Sci. Technol..

[26] Wilson MT, Hogg N, Jones GD (1990). Reactions of reduced cellobiose oxidase with oxygen. Is cellobiose oxidase primarily an oxidase?. Biochem. J..

[27] Mansfield SD, De Jong E, Saddler JN (1997). Cellobiose dehydrogenase, an active agent in cellulose depolymerization. Appl. Environ. Microbiol..

[28] Tanabe S, Ishii-Minami N, Saitoh K, Otake Y, Kaku H, Shibuya N, Nishizawa Y, Minami E (2011). The role of catalase-peroxidase secreted by *Magnaporthe oryzae* during early infection of rice cells. Mol. Plant Microbe. Interact..

[29] Yang Z, Huang J, Geng J, Nair U, Klionsky DJ (2006). Atg22 recycles amino acids to link the degradative and recycling functions of autophagy. Mol. Biols Cell..

[30] Zhang YM, Chen H, He CL, Wang Q (2013). Nitrogen starvation induced oxidative stress in an oil-producing green alga *Chlorella sorokiniana* C3. PLoS ONE.

[31] Kim S, Lee SJ, Nai YS, Yu JS, Lee MR, Yang YT, Kim JS (2016). Characterization of T-DNA insertion mutants with decreased virulence in the entomopathogenic fungus *Beauveria bassiana* JEF-007. Appl. Microbiol. Biotechnol..

[32] Ying SH, Feng MG (2019). Insight into vital role of autophagy in sustaining biological control potential of fungal pathogens against pest insects and nematodes. Virulence..

[33] Qiu L, Zhang J, Song JZ, Hu SJ, Zhang TS, Li Z, Wang JJ, Cheng W (2021). Involvement of BbTpc1, an important Zn (II) 2Cys6 transcriptional regulator, in chitin biosynthesis, fungal development and virulence of an insect mycopathogen. Int. J. Biol. Macromol..

[34] Huang S, Keyhani NO, Zhao X, Zhang Y (2019). The Thm1 Zn (II) 2Cys6 transcription factor contributes to heat, membrane integrity and virulence in the insect pathogenic fungus *Beauveria bassiana*. Environ. Microbiol..

[35] Hagiwara D, Miura D, Shimizu K, Paul S, Ohba A, Gonoi T, Watanabe A, Kamei K, Shintani T, Moye-Rowley WS, Kawamoto S, Gomi KA (2017). A novel Zn2-Cys6 transcription factor AtrR plays a key role in an azole resistance mechanism of *Aspergillus fumigatus* by co-regulating *cyp51A* and *cdr1B* expressions. PLoS Pathog..

[36] Jordá T, Puig S (2020). Regulation of ergosterol biosynthesis in *Saccharomyces cerevisiae*. Genes.

[37] Wiseman H (1993). Vitamin D is a membrane antioxidant Ability to inhibit iron-dependent lipid peroxidation in liposomes compared to cholesterol, ergosterol and tamoxifen and relevance to anticancer action. FEBS Lett..

[38] Higgins VJ, Beckhouse AG, Oliver AD, Rogers PJ, Dawes IW (2003). Yeast genome-wide expression analysis identifies a strong ergosterol and oxidative stress response during the initial stages of an industrial lager fermentation. Appl. Environ. Microbiol..

[39] Zhang J, Li X, Olmedo M, Holdorf AD, Shang Y, Artal-Sanz M, Yilmaz LS, Walhout AJ (2019). A delicate balance between bacterial iron and reactive oxygen species supports optimal *C. elegans* development. Cell Host Microbe..

[40] Philpott CC, Leidgens S, Frey AG (2012). Metabolic remodeling in iron-deficient fungi. Biochim. Biophys. Acta. Mol. Cell Res..

[41] Haurie V, Boucherie H, Sagliocco F (2003). The Snf1 protein kinase controls the induction of genes of the iron uptake pathway at the diauxic shift *in Saccharomyces cerevisiae*. J. Biol. Chem..

[42] De Groot MJ, Bundock P, Hooykaas PJ, Beijersbergen AG (1998). *Agrobacterium tumefaciens*-mediated transformation of filamentous fungi. Nat. Biotechnol..

[43] Srisuksam C, Toopaang W, Phonghanpot S, Punya J, Wattanachaisareekula S, Tanticharoen M, Cheevadhanarak S, Amnuaykanjanasin A (2015). Enhanced efficiency of Agrobacterium-mediated transformation in *Beauveria bassiana*. Proceedings of the Burapha University International Conference, Chonburi, Thailand..

[44] Konetschny-Rapp S, Huschka HG, Winkelmann G, Jung G (1988). High-performance liquid chromatography of siderophores from fungi. Biol. Met..

[45] Trapnell C, Hendrickson DG, Sauvageau M, Goff L, Rinn JL, Pachter L (2013). Differential analysis of gene regulation at transcript resolution with RNA-seq. Nat Biotechnol..

[46] Holt C, Yandell M (2011). MAKER2: an annotation pipeline and genome-database management tool for second-generation genome projects. BMC Bioinform..

[47] Goff, L., Trapnell, C., Kelley, D., 2020. CummeRbund: Analysis, exploration, manipulation, and visualization of Cufflinks high-throughput sequencing data. R package version 2.30.0.

[48] Szklarczyk D, Gable AL, Lyon D, Junge A, Wyder S, Huerta-Cepas J, Simonovic M, Doncheva NT, Morris JH, Bork P, Jensen LJ, Mering CV (2019). STRING v11: protein-protein association networks with increased coverage, supporting functional discovery in genome-wide experimental datasets. Nucleic. Acids. Res..

[49] Amnuaykanjanasin A, Jirakkakul J, Panyasiri C, Panyarakkit P, Nounurai P, Chantasingh D, Eurwilaichitr L, Cheevadhanarak S, Tanticharoen M (2013). Infection and colonization of tissues of the aphid *Myzus persicae* and cassava mealybug *Phenacoccus manihoti* by the fungus *Beauveria bassiana*. Biocontrol.

