# Supplementary material for: Iron homeostasis in the absence of ferricrocin and its consequences in fungal development and insect virulence in Beauveria bassiana
Source: Sci Rep. 2021 Oct 4;11:19624. doi: 10.1038/s41598-021-99030-4 (PMC8490459; doi:10.1038/s41598-021-99030-4)
Supplement: Supplementary file 1 — Supplementary Information 1. [file 41598_2021_99030_MOESM1_ESM.pdf]

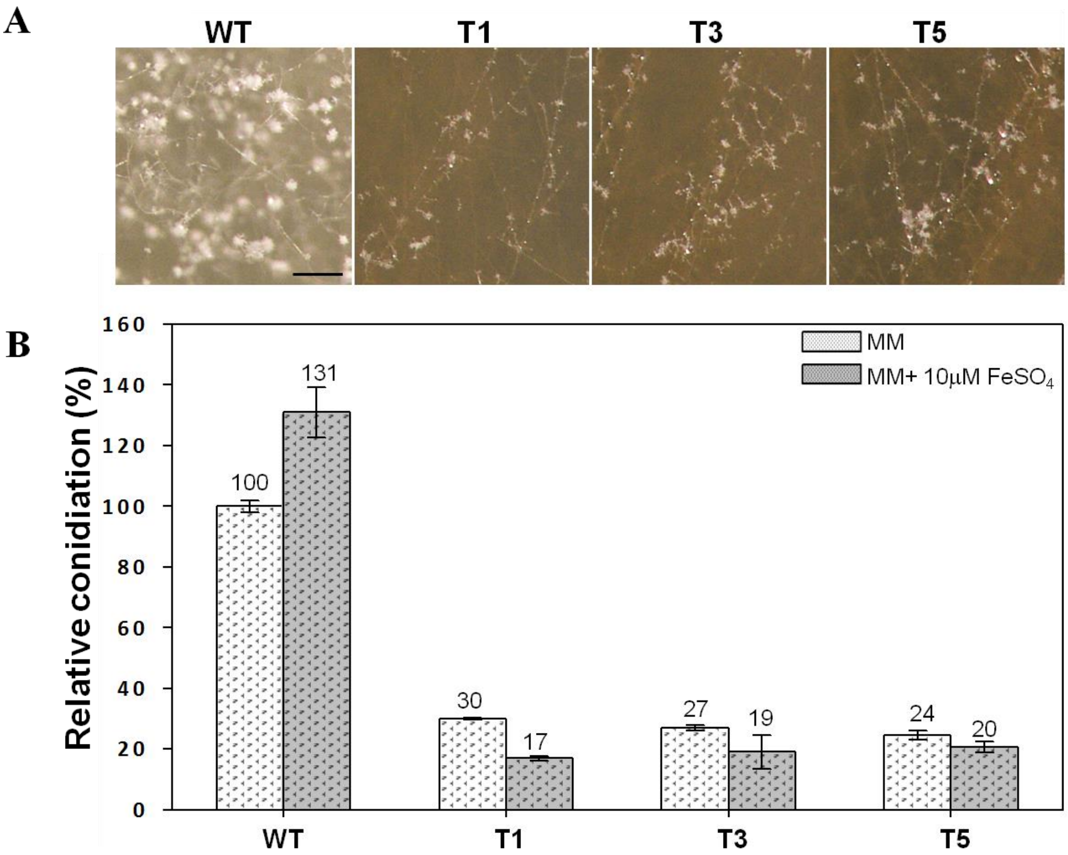

Supplemental File S1. Asexual reproduction of the ferricrocin-deficient mutants T1-T3, compared with *B. bassiana* wild type (WT). A. Different degrees of aerial hyphal formation and conidialtion were detected between the wild type colony and the mutants' colonies. Bar, 0.3 mm. **B.** Relative conidiation of *B. bassiana* wild-type and the mutants. Conidiation (%) was normalized by conidiation of the wild-type that was grown on a minimal medium. An error bar represents a standard deviation from three replicates.
