# Supplementary material for: Iron homeostasis in the absence of ferricrocin and its consequences in fungal development and insect virulence in Beauveria bassiana
Source: Sci Rep. 2021 Oct 4;11:19624. doi: 10.1038/s41598-021-99030-4 (PMC8490459; doi:10.1038/s41598-021-99030-4)
Supplement: Supplementary file 2 — Supplementary Information 2. [file 41598_2021_99030_MOESM2_ESM.pdf]

A

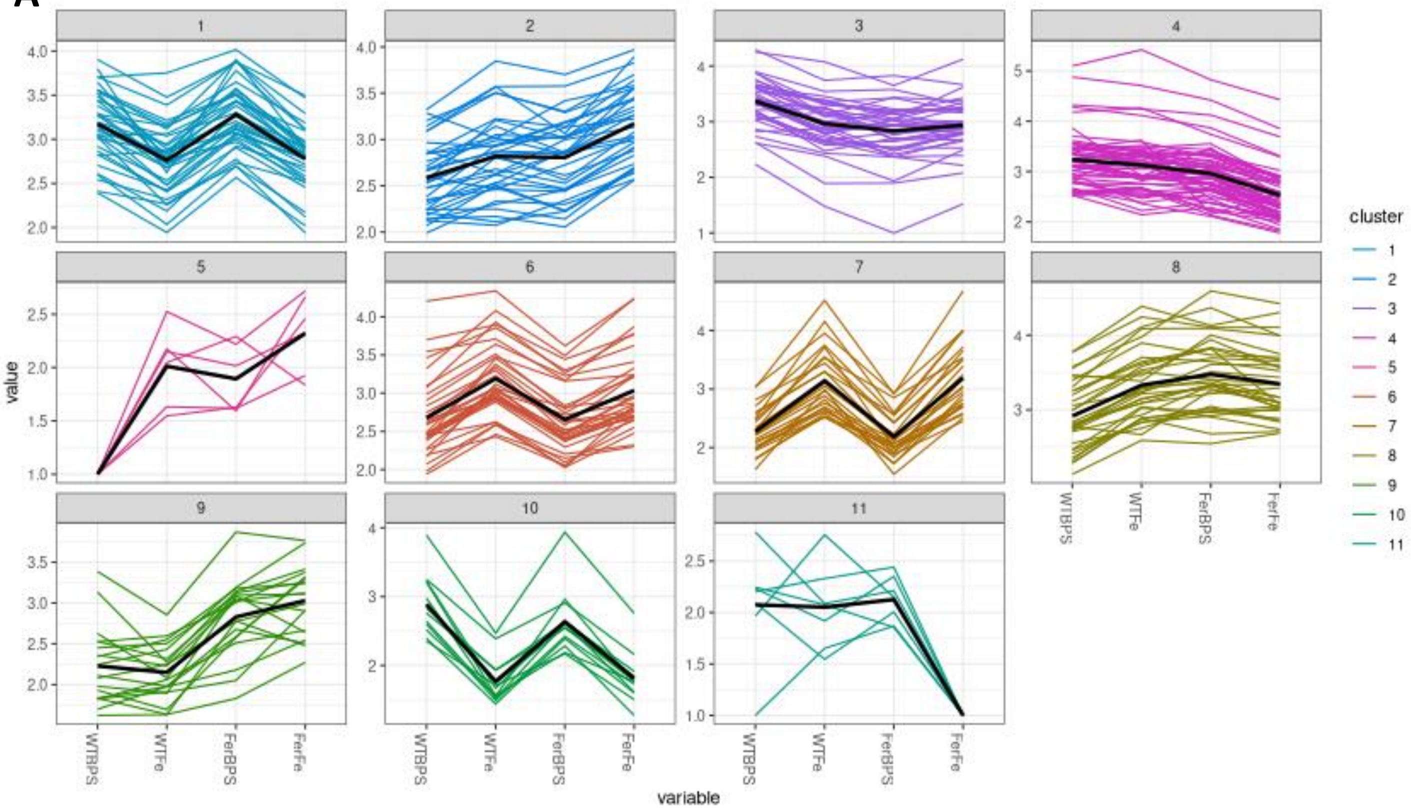

B

cluster	cluster size	WT-BPS vs Δ*ferS*-BPS	WT-BPS vs WT-Fe	Δ*ferS*-BPS vs Δ*ferS*-Fe
1	38	0	- -	- -
2	36	+	+	+
3	39	-	-	+
4	57	0	0	-
5	6	++	++	+
6	33	0	++	++
7	26	0	++	++
8	34	++	+	0
9	19	++	-	+
10	13	0	- -	- -
11	7	0	nd	- -
- -	highly negative	+	positive	
-	negative	++	highly positive	
0	neutral	nd	not described	

Supplemental File S2. DEG clustering. A. There were 11 clusters that distinguish gene expression patterns among four treatments: wild type (WT) and the mutant Δ*ferS* under iron depleted condition (WT- and Δ*ferS*-BPS) and under iron replete (WT- and Δ*ferS*-Fe). **B.** Number of genes in each cluster and comparison of gene expression between the two treatments in each pair. **WT-BPS vs Δ*ferS*-BPS**, Δ*ferS*'s higher expression over WT in iron depleted condition; **WT-BPS vs WT-Fe**, WT's higher expression in iron replete over iron depleted condition; and **Δ*ferS*-BPS vs Δ*ferS*-Fe,** Δ*ferS*'s higher expression in iron replete over iron depleted condition.
