# Supplementary material for: Iron homeostasis in the absence of ferricrocin and its consequences in fungal development and insect virulence in Beauveria bassiana
Source: Sci Rep. 2021 Oct 4;11:19624. doi: 10.1038/s41598-021-99030-4 (PMC8490459; doi:10.1038/s41598-021-99030-4)
Supplement: Supplementary file 3 — Supplementary Information 3. [file 41598_2021_99030_MOESM3_ESM.pdf]

| clustering | gene_symbol | functional_annotation | WTBPS_spec | WTFe_spec | FerBPS_spec | FerFe_spec |
|---|---|---|---|---|---|---|
| 1 | | Membrane protein, putative | 0.30505 | 0.21237 | 0.27116 | 0.24544 |
| 1 | ATG4 | cysteine protease ATG4 [EC:3.4.22.-] | 0.30264 | 0.22723 | 0.26543 | 0.23953 |
| 1 | | Pristinamycin IIA synthase subunit A | 0.30029 | 0.19873 | 0.30852 | 0.22379 |
| 1 | | Taurine catabolism dioxygenase TauD | 0.29976 | 0.21613 | 0.31439 | 0.20051 |
| 1 | | Trypsin-related protease | 0.29473 | 0.23922 | 0.26886 | 0.23251 |
| 1 | | Uncharacterized protein | 0.29205 | 0.23554 | 0.27389 | 0.23382 |
| 1 | KEX1 | carboxypeptidase D [EC:3.4.16.6] | 0.28874 | 0.23267 | 0.27387 | 0.24021 |
| 1 | TPP1, CLN2 | tripeptidyl-peptidase I [EC:3.4.14.9] | 0.28859 | 0.21777 | 0.27905 | 0.24949 |
| 1 | | Alcohol dehydrogenase GroES-like domain-containing | 0.28699 | 0.23200 | 0.27570 | 0.24081 |
| 1 | | Taurine catabolism dioxygenase TauD | 0.28682 | 0.25140 | 0.27139 | 0.22592 |
| 1 | SLC39A7, KE4, ZIP7 | ZIP Zinc transporter | 0.28589 | 0.24470 | 0.27956 | 0.22513 |
| 1 | | Methyltransferase-like protein | 0.28532 | 0.21182 | 0.29613 | 0.24070 |
| 1 | FEN2, LIZ1 | MFS transporter, ACS family, pantothenate transporte | 0.28469 | 0.21196 | 0.31115 | 0.22498 |
| 1 | | ATP-grasp enzyme-like protein | 0.28336 | 0.22895 | 0.28789 | 0.23484 |
| 1 | TPP1, CLN2 | tripeptidyl-peptidase I [EC:3.4.14.9]; Alkaline serine pr | 0.28129 | 0.23096 | 0.28065 | 0.24267 |
| 1 | | Uncharacterized protein | 0.28076 | 0.25170 | 0.27660 | 0.22657 |
| 1 | | Uncharacterized protein | 0.27875 | 0.24720 | 0.27688 | 0.23303 |
| 1 | aroB | 3-dehydroquinate synthase | 0.27690 | 0.23615 | 0.28194 | 0.24077 |
| 1 | | Uncharacterized protein | 0.27587 | 0.25009 | 0.28285 | 0.22678 |
| 1 | DEGS | Sphingolipid delta(4)-desaturase (EC 1.14.19.17) | 0.27466 | 0.25375 | 0.27813 | 0.22931 |
| 1 | uvsE, UVE1 | UV DNA damage endonuclease [EC:3.-.-.-] | 0.27435 | 0.24358 | 0.29419 | 0.22286 |
| 1 | FTR, FTH1, efeU | high-affinity iron transporter | 0.27418 | 0.23162 | 0.29687 | 0.23226 |
| 1 | | Mitochondrial carrier protein | 0.27220 | 0.21388 | 0.29877 | 0.24959 |
| 1 | | MARVEL domain-containing protein | 0.27122 | 0.24160 | 0.28062 | 0.24264 |
| 1 | SC5DL, ERG3 | Delta7-sterol 5-desaturase [EC:1.14.19.20]; Lathoster | 0.27064 | 0.23419 | 0.29667 | 0.23363 |
| 1 | | Ferulic acid esterase (FaeA), putative | 0.27035 | 0.22265 | 0.29297 | 0.24918 |
| 1 | | Uncharacterized protein | 0.26822 | 0.25658 | 0.28723 | 0.22344 |
| 1 | VVD | Vivid PAS protein VVD | 0.26590 | 0.24189 | 0.27849 | 0.25001 |
| 1 | FET3_5 | iron transport multicopper oxidase | 0.26441 | 0.22879 | 0.28618 | 0.25637 |
| 1 | RPB9, POLR2I | RNA polymerase M/15 kDa subunit | 0.26256 | 0.23964 | 0.29659 | 0.23665 |
| 1 | | Sex-determining protein fem-1 | 0.26158 | 0.24140 | 0.31123 | 0.21993 |
| 1 | | Oligopeptide transporter | 0.26015 | 0.20673 | 0.29770 | 0.26952 |
| 1 | | Protein-serine/threonine kinase (EC 2.7.11.-) | 0.25738 | 0.26236 | 0.28470 | 0.23148 |
| 1 | PHO84 | MFS transporter, PHS family, inorganic phosphate tra | 0.25727 | 0.24275 | 0.29262 | 0.24318 |
| 1 | | PH domain-containing protein | 0.25705 | 0.26042 | 0.27899 | 0.23985 |
| 1 | SCD, desC | stearoyl-CoA desaturase (Delta-9 desaturase) [EC:1.1 | 0.25700 | 0.23523 | 0.29910 | 0.24407 |

| | | | | | | |
|---|---|---|---|---|---|---|
| 1 | SUV39H, CLR4 | [histone H3]-lysine9 N-trimethyltransferase SUV39H [I | 0.25467 | 0.25688 | 0.29102 | 0.23320 |
| 1 | | Extradiol ring-cleavage dioxygenase class III protein s | 0.25429 | 0.22224 | 0.29436 | 0.26430 |
| 2 | | Uncharacterized protein | 0.25618 | 0.22978 | 0.26825 | 0.28168 |
| 2 | | DUF300 domain protein, putative | 0.25591 | 0.23246 | 0.26704 | 0.28062 |
| 2 | MAN1A_C, MNS1_2 | mannosyl-oligosaccharide alpha-1,2-mannosidase [EC | 0.24680 | 0.23147 | 0.26188 | 0.29532 |
| 2 | | Pyridine nucleotide-disulfide oxidoreductase, putative | 0.24596 | 0.24676 | 0.23033 | 0.31154 |
| 2 | | Homeobox and C2H2 transcription factor, putative | 0.24525 | 0.23013 | 0.24738 | 0.31180 |
| 2 | OefC | C6 transcription factor OefC | 0.24509 | 0.24901 | 0.23040 | 0.31017 |
| 2 | SUOX | sulfite oxidase [EC:1.8.3.1] | 0.24461 | 0.24049 | 0.26420 | 0.28669 |
| 2 | | 2-nitropropane dioxygenase | 0.24101 | 0.26521 | 0.24908 | 0.28092 |
| 2 | | X-Pro dipeptidyl-peptidase (S15 family) protein | 0.24023 | 0.25725 | 0.23967 | 0.29832 |
| 2 | CYP645A1 | Cytochrome P450 CYP645A1 | 0.23662 | 0.26126 | 0.24317 | 0.29455 |
| 2 | MRS3, SLC25A28_37, | Mitochondrial RNA-splicing protein MRS3 | 0.23642 | 0.27097 | 0.25334 | 0.27546 |
| 2 | ACSL, fadD | Long-chain-fatty-acid-CoA ligase | 0.23520 | 0.26780 | 0.23759 | 0.29480 |
| 2 | | Cellobiose dehydrogenase, putative | 0.23476 | 0.24880 | 0.24613 | 0.30539 |
| 2 | | Choline/Carnitine O-acyltransferase | 0.23474 | 0.24361 | 0.26717 | 0.29016 |
| 2 | | Acyl-CoA dehydrogenase | 0.23375 | 0.25598 | 0.25947 | 0.28674 |
| 2 | | CoA-transferase family III | 0.23299 | 0.27260 | 0.23099 | 0.29832 |
| 2 | ABCG2.PDR, CDR1 | ATP-binding cassette, subfamily G (WHITE), member | 0.23297 | 0.25520 | 0.25283 | 0.29461 |
| 2 | | Putative Zn(II)2Cys6 transcription factor | 0.23276 | 0.22507 | 0.26632 | 0.31003 |
| 2 | | Monodehydroascorbate reductase | 0.23058 | 0.26905 | 0.25860 | 0.27778 |
| 2 | | Protein family CysZ | 0.23001 | 0.24756 | 0.27462 | 0.28350 |
| 2 | BCS1 | BCS1 protein | 0.22970 | 0.24609 | 0.27275 | 0.28704 |
| 2 | | Uncharacterized protein | 0.22863 | 0.26241 | 0.26285 | 0.28199 |
| 2 | FET4 | Low affinity iron transporter | 0.22847 | 0.27223 | 0.23505 | 0.29910 |
| 2 | E4.1.3.1, aceA | isocitrate lyase [EC:4.1.3.1] | 0.22626 | 0.26025 | 0.25225 | 0.29653 |
| 2 | FAH, fahA | fumarylacetoacetase [EC:3.7.1.2] | 0.22579 | 0.26434 | 0.26094 | 0.28461 |
| 2 | CS, gltA | citrate synthase [EC:2.3.3.1] | 0.22474 | 0.25849 | 0.26050 | 0.29170 |
| 2 | | RadR putative transcriptional regulator | 0.22471 | 0.26644 | 0.23914 | 0.30435 |
| 2 | ilvD | Dihydroxy-acid dehydratase | 0.22423 | 0.26468 | 0.24549 | 0.30054 |
| 2 | | Amidase family protein | 0.22182 | 0.26173 | 0.25232 | 0.29911 |
| 2 | CSR1 | Phosphatidylinositol transfer protein CSR1 | 0.21983 | 0.24433 | 0.26625 | 0.30411 |
| 2 | | Uncharacterized protein | 0.21641 | 0.23948 | 0.28056 | 0.29783 |
| 2 | | Cutinase transcription factor 1 beta | 0.21430 | 0.27787 | 0.23845 | 0.30334 |
| 2 | | ABC transporter | 0.21116 | 0.26445 | 0.25957 | 0.29921 |
| 2 | serB, PSPH | Phosphoserine phosphatase | 0.21067 | 0.27932 | 0.25650 | 0.28806 |
| 2 | | Putative cell wall glycoprotein | 0.20770 | 0.27082 | 0.25300 | 0.30242 |

| | | | | | | |
|---|---|---|---|---|---|---|
| 2 | CYP542B3 | Cytochrome P450 CYP542B3 | 0.20144 | 0.26040 | 0.27022 | 0.30147 |
| 3 | | CVNH domain-containing protein | 0.47923 | 0.23090 | 0.00000 | 0.24602 |
| 3 | | FAD binding domain containing protein | 0.34336 | 0.21755 | 0.21855 | 0.25156 |
| 3 | RP-S28e, RPS28 | 40S ribosomal protein S28 | 0.32409 | 0.25583 | 0.20912 | 0.24375 |
| 3 | | Uncharacterized protein | 0.31635 | 0.25714 | 0.21673 | 0.24352 |
| 3 | HGD, hmgA | homogentisate 1,2-dioxygenase [EC:1.13.11.5] | 0.30724 | 0.22647 | 0.25238 | 0.24867 |
| 3 | | SelT/selW/selH selenoprotein domain-containing prot | 0.30320 | 0.24716 | 0.24207 | 0.24285 |
| 3 | SLC35B1 | solute carrier family 35 (UDP-galactose transporter), r | 0.29998 | 0.24443 | 0.23014 | 0.26065 |
| 3 | PET309 | pentatricopeptide repeat-containing protein PET309; ( | 0.29996 | 0.24932 | 0.24376 | 0.24243 |
| 3 | | Uncharacterized protein | 0.29896 | 0.25869 | 0.24948 | 0.22810 |
| 3 | | Clathrin-coated vesicle protein, putative | 0.29665 | 0.24069 | 0.23640 | 0.26171 |
| 3 | ACLH | RadH flavin-dependent halogenase | 0.29595 | 0.26297 | 0.19539 | 0.27878 |
| 3 | | Putative phospholipase (EC 3.1.1.47) | 0.29582 | 0.23114 | 0.25592 | 0.25262 |
| 3 | | Uncharacterized protein | 0.29503 | 0.26941 | 0.23402 | 0.23684 |
| 3 | | AhpC-TSA domain-containing protein | 0.29486 | 0.24235 | 0.25745 | 0.24103 |
| 3 | | Cys/Met metabolism PLP-dependent enzyme | 0.29392 | 0.23981 | 0.24320 | 0.25879 |
| 3 | | Thioredoxin-like protein | 0.29387 | 0.25380 | 0.22871 | 0.25913 |
| 3 | PRO1, NOSA | C6 transcription factor Pro1 | 0.29375 | 0.25378 | 0.24656 | 0.24172 |
| 3 | PFN | Profilin | 0.29320 | 0.24803 | 0.23671 | 0.25779 |
| 3 | | Sulfate transporter | 0.29277 | 0.23231 | 0.25793 | 0.25266 |
| 3 | PPWD1, CLD | Cyclophilin type peptidyl-prolyl cis-trans isomerase/CL | 0.29131 | 0.24519 | 0.25229 | 0.24717 |
| 3 | CHMP5, VPS60 | charged multivesicular body protein 5 | 0.28981 | 0.24007 | 0.25015 | 0.25594 |
| 3 | | DUF500 domain protein | 0.28978 | 0.25318 | 0.23264 | 0.26019 |
| 3 | | Eukaryotic initiation factor 4E | 0.28925 | 0.24281 | 0.25015 | 0.25382 |
| 3 | VPS24, CHMP3 | charged multivesicular body protein 3 | 0.28859 | 0.25545 | 0.24491 | 0.24712 |
| 3 | TAN1, THUMPD1 | tRNA acetyltransferase TAN1; THUMP domain-contai | 0.28859 | 0.23785 | 0.25269 | 0.25685 |
| 3 | | Uncharacterized protein | 0.28665 | 0.24965 | 0.25559 | 0.24426 |
| 3 | | DnaJ domain-containing protein | 0.28540 | 0.25567 | 0.24539 | 0.24975 |
| 3 | | Transcriptional activator | 0.28489 | 0.24955 | 0.26298 | 0.23865 |
| 3 | clpB | ATP-dependent Clp protease ATP-binding subunit Clp | 0.28413 | 0.28157 | 0.22286 | 0.24664 |
| 3 | | Phosphoinositide phospholipase C (EC 3.1.4.11) | 0.28311 | 0.26754 | 0.24798 | 0.23741 |
| 3 | | Uncharacterized protein | 0.28289 | 0.25651 | 0.25900 | 0.23776 |
| 3 | | Uncharacterized protein | 0.28038 | 0.26638 | 0.24738 | 0.24209 |
| 3 | | Uncharacterized protein | 0.27998 | 0.26172 | 0.24157 | 0.25304 |
| 3 | ATPeV0D, ATP6D | V-type proton ATPase subunit | 0.27954 | 0.25501 | 0.26082 | 0.24095 |
| 3 | | Helix-loop-helix DNA-binding domain-containing prote | 0.27442 | 0.28069 | 0.22091 | 0.25942 |
| 3 | PPID, CYPD | Cyclophilin type peptidyl-prolyl cis-trans isomerase/CL | 0.27396 | 0.27792 | 0.23064 | 0.25340 |

| | | | | | | |
|---|---|---|---|---|---|---|
| 3 | RP-L34e, RPL34 | large subunit ribosomal protein L34e | 0.27383 | 0.26240 | 0.23462 | 0.26541 |
| 3 | | Uncharacterized protein | 0.27001 | 0.25881 | 0.23011 | 0.27705 |
| 3 | | Transmembrane amino acid transporter | 0.26969 | 0.28454 | 0.23469 | 0.24697 |
| 4 | | Uncharacterized protein | 0.35335 | 0.26820 | 0.22981 | 0.17571 |
| 4 | cynT, can | Carbonic anhydrase (EC 4.2.1.1) (Carbonate dehydra | 0.31901 | 0.23410 | 0.28545 | 0.19257 |
| 4 | | Hisactophilin C49S mutant/phototropin PHY3 fusion p | 0.31673 | 0.27216 | 0.25202 | 0.19085 |
| 4 | | Volvatoxin A2 | 0.31306 | 0.29435 | 0.21948 | 0.20470 |
| 4 | | Amino acid permease | 0.31105 | 0.24938 | 0.27311 | 0.19924 |
| 4 | | Uncharacterized protein | 0.30967 | 0.25275 | 0.27608 | 0.19387 |
| 4 | | Heat shock protein 30 | 0.30927 | 0.32403 | 0.17371 | 0.22024 |
| 4 | | MFS transporter, putative | 0.30851 | 0.24321 | 0.26797 | 0.21428 |
| 4 | | Uncharacterized protein | 0.30818 | 0.28905 | 0.24534 | 0.18898 |
| 4 | | Major facilitator superfamily, general substrate transpo | 0.30725 | 0.28715 | 0.26207 | 0.17357 |
| 4 | | Dynamin GTPase | 0.30522 | 0.30933 | 0.23315 | 0.18212 |
| 4 | | Glycosyl hydrolase | 0.30042 | 0.29599 | 0.25730 | 0.17672 |
| 4 | | Uncharacterized protein | 0.29872 | 0.28374 | 0.24587 | 0.20524 |
| 4 | CDC6 | Cell division control protein | 0.29709 | 0.25100 | 0.27680 | 0.20925 |
| 4 | | Adhesin protein Mad2 | 0.29639 | 0.28018 | 0.25788 | 0.19891 |
| 4 | | DNA mismatch repair protein msh-2 | 0.29251 | 0.25660 | 0.27047 | 0.21525 |
| 4 | | Uncharacterized protein | 0.29231 | 0.28083 | 0.24126 | 0.22033 |
| 4 | | Major facilitator superfamily transporter | 0.29220 | 0.28189 | 0.26694 | 0.19178 |
| 4 | CYP625A1 | Cytochrome P450 CYP625A1 | 0.29123 | 0.30061 | 0.23583 | 0.20536 |
| 4 | | HET domain-containing protein | 0.29082 | 0.25592 | 0.27653 | 0.21128 |
| 4 | | Protein arv1 | 0.28965 | 0.27342 | 0.26159 | 0.20989 |
| 4 | | Uncharacterized protein | 0.28902 | 0.27980 | 0.25543 | 0.21022 |
| 4 | | Uncharacterized protein | 0.28891 | 0.26932 | 0.26272 | 0.21394 |
| 4 | HSL1 | BR serine/threonine-protein kinase | 0.28823 | 0.26805 | 0.25339 | 0.22586 |
| 4 | | Uncharacterized protein | 0.28609 | 0.25837 | 0.26840 | 0.22260 |
| 4 | abf1 | Arabinofuranosidase B | 0.28527 | 0.26716 | 0.25112 | 0.23231 |
| 4 | GYS | Glycogen [starch] synthase (EC 2.4.1.11) | 0.28520 | 0.25877 | 0.26132 | 0.23059 |
| 4 | | Uncharacterized protein | 0.28509 | 0.27415 | 0.25751 | 0.21845 |
| 4 | | Uncharacterized protein | 0.28359 | 0.30520 | 0.25194 | 0.19146 |
| 4 | AXL2 | axial budding pattern protein 2 | 0.28329 | 0.27245 | 0.25729 | 0.22245 |
| 4 | | Glycoside hydrolase family 55 | 0.28243 | 0.29896 | 0.23779 | 0.21486 |
| 4 | | Uncharacterized protein | 0.28227 | 0.27290 | 0.25664 | 0.22376 |
| 4 | | Beta-galactosidase (EC 3.2.1.23) | 0.28180 | 0.27620 | 0.27277 | 0.20340 |
| 4 | | Spa2-like protein | 0.28055 | 0.25721 | 0.27235 | 0.22560 |

| | | | | | | |
|---|---|---|---|---|---|---|
| 4 | | Mmc protein | 0.28029 | 0.28572 | 0.25020 | 0.21882 |
| 4 | | Cyclin domain-containing protein | 0.28027 | 0.27026 | 0.25927 | 0.22595 |
| 4 | alc, ALLC | Allantoicase-like protein | 0.28002 | 0.26261 | 0.26019 | 0.23328 |
| 4 | | Glutaminase GtaA | 0.27978 | 0.27563 | 0.26086 | 0.21907 |
| 4 | | Uncharacterized protein | 0.27628 | 0.26971 | 0.27647 | 0.21250 |
| 4 | | Uncharacterized protein | 0.27613 | 0.26455 | 0.26757 | 0.22767 |
| 4 | | Pumilio domain-containing protein | 0.27590 | 0.27241 | 0.26098 | 0.22655 |
| 4 | | PLC-like phosphodiesterase | 0.27585 | 0.27285 | 0.27255 | 0.21382 |
| 4 | | Allergen-like protein | 0.27510 | 0.26024 | 0.27596 | 0.22441 |
| 4 | | Endoplasmic Reticulum Oxidoreductin 1 | 0.27463 | 0.26795 | 0.26977 | 0.22336 |
| 4 | | Uncharacterized protein | 0.27435 | 0.27248 | 0.28537 | 0.20178 |
| 4 | TEAD | transcriptional enhancer factor | 0.27385 | 0.27545 | 0.26158 | 0.22488 |
| 4 | ENDOG | endonuclease G, mitochondrial | 0.27384 | 0.27087 | 0.27527 | 0.21521 |
| 4 | | WSC domain-containing protein | 0.27359 | 0.32450 | 0.24481 | 0.18800 |
| 4 | | Uncharacterized protein | 0.27352 | 0.26851 | 0.26099 | 0.23315 |
| 4 | | Eukaryotic aspartyl protease | 0.27338 | 0.29114 | 0.25412 | 0.21625 |
| 4 | LYER | cell growth-regulating nucleolar protein; zf-LYAR doma | 0.27214 | 0.27172 | 0.26168 | 0.23053 |
| 4 | | Uncharacterized protein | 0.27149 | 0.27384 | 0.28415 | 0.20479 |
| 4 | CTU2, NCS2 | Cytoplasmic tRNA 2-thiolation protein 2 | 0.26901 | 0.27772 | 0.26306 | 0.22603 |
| 4 | GAOA | Galactose oxidase | 0.26869 | 0.27626 | 0.26918 | 0.22146 |
| 4 | | Hydrophobin | 0.26691 | 0.28268 | 0.25324 | 0.23315 |
| 4 | LDB19, ART1 | LDB19 domain-containing protein | 0.26548 | 0.28446 | 0.25799 | 0.22784 |
| 4 | hmp, YHB1 | nitric oxide dioxygenase [EC:1.14.12.17]; Flavohemop | 0.25825 | 0.29067 | 0.24930 | 0.23765 |
| 5 | | Uncharacterized protein | 0.00000 | 0.31979 | 0.37597 | 0.26938 |
| 5 | | Uncharacterized protein | 0.00000 | 0.31925 | 0.29087 | 0.35630 |
| 5 | SIW14 | Tyrosine-protein phosphatase SIW14 | 0.00000 | 0.32857 | 0.27710 | 0.36020 |
| 5 | | Uncharacterized protein | 0.00000 | 0.29044 | 0.28525 | 0.38925 |
| 5 | | Uncharacterized protein | 0.00000 | 0.32918 | 0.19613 | 0.43112 |
| 5 | | DJ-1/PfpI family protein | 0.00000 | 0.22538 | 0.24977 | 0.48069 |
| 6 | | Uncharacterized protein | 0.26580 | 0.27421 | 0.22828 | 0.26768 |
| 6 | RP-L14e, RPL14 | large subunit ribosomal protein L14e | 0.25902 | 0.27313 | 0.24026 | 0.26402 |
| 6 | CYP52G8 | Cytochrome P450 CYP52G8 | 0.25702 | 0.26955 | 0.22755 | 0.28167 |
| 6 | PES1, NOP7 | Pescadillo homolog (Nucleolar protein 7 homolog) | 0.25520 | 0.28679 | 0.22694 | 0.26672 |
| 6 | E2.6.1.42, ilvE | branched-chain amino acid aminotransferase [EC:2.6 | 0.25458 | 0.27614 | 0.19648 | 0.30563 |
| 6 | | Major facilitator superfamily transporter | 0.25326 | 0.30069 | 0.21624 | 0.26442 |
| 6 | RP-S16e, RPS16 | small subunit ribosomal protein S16e | 0.25165 | 0.28152 | 0.23902 | 0.26400 |
| 6 | ACO2 | Aconitate hydratase, mitochondrial (Aconitase) (EC 4. | 0.25060 | 0.30224 | 0.22128 | 0.26068 |

| | | | | | | |
|---|---|---|---|---|---|---|
| 6 | LYS21, LYS20 | homocitrate synthase [EC:2.3.3.14] | 0.24380 | 0.29102 | 0.22550 | 0.27488 |
| 6 | | NlpC/P60-like cell-wall peptidase, putative | 0.24322 | 0.31119 | 0.23565 | 0.24463 |
| 6 | | Arginase-like protein | 0.24285 | 0.28194 | 0.22670 | 0.28382 |
| 6 | | Uncharacterized protein | 0.24182 | 0.29287 | 0.21720 | 0.28263 |
| 6 | LYS2 | L-2-aminoadipate reductase [EC:1.2.1.95] | 0.24180 | 0.29912 | 0.22488 | 0.26911 |
| 6 | | Alcohol dehydrogenase | 0.23883 | 0.28707 | 0.22133 | 0.28749 |
| 6 | | N-acetyl-gamma-glutamyl-phosphate reductase | 0.23832 | 0.28358 | 0.24949 | 0.26470 |
| 6 | asnB, ASNS | asparagine synthase (glutamine-hydrolysing) [EC:6.3. | 0.23831 | 0.29991 | 0.22975 | 0.26704 |
| 6 | cysI | sulfite reductase (NADPH) hemoprotein beta-compon | 0.23685 | 0.29168 | 0.22676 | 0.27968 |
| 6 | LYS4 | homoaconitate hydratase [EC:4.2.1.36] | 0.23683 | 0.29158 | 0.23348 | 0.27346 |
| 6 | | C6 zinc finger domain protein | 0.23681 | 0.27221 | 0.21622 | 0.30862 |
| 6 | cysQ, MET22, BPNT1 | 3',5'-bisphosphate nucleotidase | 0.23306 | 0.28375 | 0.25595 | 0.26323 |
| 6 | lysC | aspartate kinase [EC:2.7.2.4] | 0.23175 | 0.29598 | 0.24604 | 0.26169 |
| 6 | SNW1, SKIIP, SKIP | SKIP/SNW domain-containing protein | 0.23033 | 0.28140 | 0.25560 | 0.26858 |
| 6 | katG | catalase-peroxidase [EC:1.11.1.21] | 0.22597 | 0.27985 | 0.23831 | 0.29088 |
| 6 | DARS | aspartyl-tRNA synthetase [EC:6.1.1.12] | 0.22552 | 0.28633 | 0.25329 | 0.27041 |
| 6 | LEU1 | 3-isopropylmalate dehydratase [EC:4.2.1.33] | 0.22519 | 0.30295 | 0.21842 | 0.28695 |
| 6 | | WW domain-containing protein | 0.22397 | 0.29124 | 0.23956 | 0.28016 |
| 6 | leuA, IMS | 2-isopropylmalate synthase [EC:2.3.3.13] | 0.22246 | 0.29971 | 0.23389 | 0.27835 |
| 6 | sat, met3 | Sulfate adenylyltransferase (EC 2.7.7.4) (ATP-sulfuryl | 0.21894 | 0.30794 | 0.23446 | 0.27263 |
| 6 | AFG2, DRG1, SPATA5 | AAA family ATPase | 0.21371 | 0.29841 | 0.24905 | 0.27325 |
| 6 | | C6 transcription factor, putative | 0.21266 | 0.30645 | 0.24841 | 0.26655 |
| 6 | cysK | cysteine synthase [EC:2.5.1.47] | 0.19989 | 0.30553 | 0.24535 | 0.28211 |
| 6 | GINS2, PSF2 | GINS complex subunit 2 | 0.19773 | 0.31018 | 0.25484 | 0.27005 |
| 7 | ABCB1, CD243 | ATP-binding cassette, subfamily B (MDR/TAP), memb | 0.23696 | 0.29630 | 0.19646 | 0.30237 |
| 7 | cypD_E, CYP102A2_3 | Bifunctional cytochrome P450/NADPH--P450 reducta | 0.22960 | 0.30622 | 0.17734 | 0.31560 |
| 7 | NAALAD | N-acetylated-alpha-linked acidic dipeptidase [EC:3.4.1 | 0.22555 | 0.30214 | 0.21197 | 0.29331 |
| 7 | | Acyl-CoA N-acyltransferase | 0.22378 | 0.29576 | 0.21460 | 0.29895 |
| 7 | | Oxidoreductase, 2-nitropropane dioxygenase family, p | 0.21763 | 0.30421 | 0.18072 | 0.32553 |
| 7 | AOC3, AOC2, tynA | Amine oxidase (EC 1.4.3.-) | 0.21735 | 0.30887 | 0.18333 | 0.31899 |
| 7 | | AMP-binding enzyme | 0.21710 | 0.30927 | 0.20587 | 0.29931 |
| 7 | ABCB1, CD243 | ATP-binding cassette, subfamily B (MDR/TAP), memb | 0.21570 | 0.30690 | 0.18771 | 0.31871 |
| 7 | | Bifunctional transcriptional activator/DNA repair enzym | 0.21554 | 0.30144 | 0.17146 | 0.33770 |
| 7 | | Secretory lipase | 0.21544 | 0.30885 | 0.20015 | 0.30628 |
| 7 | | Uncharacterized protein | 0.21183 | 0.37393 | 0.17556 | 0.26306 |
| 7 | | Nonribosomal peptide synthetase 10 | 0.21141 | 0.29290 | 0.20990 | 0.31710 |
| 7 | CYP52T1 | Cytochrome P450 CYP52T1 | 0.21091 | 0.28513 | 0.21781 | 0.31811 |

| | | | | | | |
|---|---|---|---|---|---|---|
| 7 | SIDL, iucB | N5-hydroxyornithine acetyltransferase [EC:2.3.1.-]; Ae | 0.21036 | 0.27568 | 0.20665 | 0.33748 |
| 7 | | Thioesterase-like protein | 0.20898 | 0.30917 | 0.20742 | 0.30532 |
| 7 | | NAD-binding Rossmann fold oxidoreductase, putative | 0.20696 | 0.30608 | 0.20164 | 0.31528 |
| 7 | VIT | vacuolar iron transporter family protein | 0.20381 | 0.30941 | 0.19536 | 0.32016 |
| 7 | | Amidohydrolase family protein | 0.20323 | 0.32349 | 0.16107 | 0.33508 |
| 7 | | Uncharacterized protein | 0.20307 | 0.31403 | 0.20301 | 0.30957 |
| 7 | E3.5.1.4, amiE | Fatty-acid amide hydrolase | 0.20171 | 0.30600 | 0.21670 | 0.30663 |
| 7 | | Penicillin-binding protein | 0.19858 | 0.29878 | 0.23577 | 0.29914 |
| 7 | pvdA, SIDA | L-ornithine N5-monooxygenase [EC:1.14.13.195 1.14 | 0.19363 | 0.29068 | 0.19386 | 0.34841 |
| 7 | ACP7 | acid phosphatase type 7; Purple acid phosphatase (E | 0.18996 | 0.30552 | 0.22051 | 0.31386 |
| 7 | CYP52T1 | Cytochrome P450 CYP52T1 | 0.18754 | 0.31736 | 0.17661 | 0.34191 |
| 7 | | Zn-dependent alcohol dehydrogenases (ISS) | 0.18637 | 0.32794 | 0.19601 | 0.31607 |
| 7 | | Amine oxidase | 0.16165 | 0.38945 | 0.14544 | 0.31499 |
| 8 | | Uncharacterized protein | 0.24703 | 0.24860 | 0.28446 | 0.25616 |
| 8 | | DUF1014 domain protein | 0.24558 | 0.23931 | 0.28478 | 0.26635 |
| 8 | AKR1A1, adh | Alcohol dehydrogenase GroES-like domain-containing | 0.24111 | 0.23033 | 0.28973 | 0.27423 |
| 8 | H2A | Histone H2A | 0.24080 | 0.27119 | 0.26221 | 0.26228 |
| 8 | RP-LP1, RPLP1 | large subunit ribosomal protein LP1 | 0.23788 | 0.27469 | 0.26297 | 0.26080 |
| 8 | | CAP22 protein | 0.23554 | 0.27385 | 0.25803 | 0.26882 |
| 8 | | Uncharacterized protein | 0.23512 | 0.26127 | 0.28413 | 0.25553 |
| 8 | | Mpv17/PMP22 family protein | 0.23488 | 0.26309 | 0.27844 | 0.25978 |
| 8 | | Uncharacterized protein | 0.23394 | 0.27349 | 0.26922 | 0.25954 |
| 8 | map | methionyl aminopeptidase [EC:3.4.11.18] | 0.23280 | 0.26259 | 0.27649 | 0.26427 |
| 8 | uraH, pucM, hiuH | 5-hydroxyisourate hydrolase [EC:3.5.2.17] | 0.23265 | 0.24747 | 0.27833 | 0.27739 |
| 8 | UCHL3, YUH1 | ubiquitin carboxyl-terminal hydrolase L3 [EC:3.4.19.12 | 0.23177 | 0.27513 | 0.26713 | 0.26208 |
| 8 | | Putative Zn(II)2Cys6 transcription factor | 0.23155 | 0.25390 | 0.28534 | 0.26508 |
| 8 | PDK2_3_4 | pyruvate dehydrogenase kinase 2/3/4 [EC:2.7.11.2] | 0.23037 | 0.25256 | 0.29079 | 0.26195 |
| 8 | | BTB/POZ domain protein | 0.23035 | 0.25842 | 0.27003 | 0.27719 |
| 8 | LYS1 | Saccharopine dehydrogenase [NAD(+), L-lysine-formi | 0.22913 | 0.26609 | 0.28242 | 0.25824 |
| 8 | | Uncharacterized protein | 0.22850 | 0.26195 | 0.27426 | 0.27125 |
| 8 | | Uncharacterized protein | 0.22774 | 0.27727 | 0.29627 | 0.23350 |
| 8 | | Zn(2)-C6 fungal-type domain-containing protein | 0.22750 | 0.27111 | 0.27716 | 0.26010 |
| 8 | | WSC domain-containing protein | 0.22472 | 0.25838 | 0.27571 | 0.27688 |
| 8 | HOGA1 | 4-hydroxy-2-oxoglutarate aldolase [EC:4.1.3.16] | 0.22418 | 0.26484 | 0.28040 | 0.26627 |
| 8 | FRG1 | protein FRG1 | 0.22377 | 0.27056 | 0.28128 | 0.26001 |
| 8 | CENPI | centromere protein I; Mis6 domain-containing protein | 0.22353 | 0.28651 | 0.27185 | 0.25355 |
| 8 | COA4 | cytochrome c oxidase assembly factor 4; CHCH doma | 0.22332 | 0.24881 | 0.28680 | 0.27637 |

| | | | | | | |
|---|---|---|---|---|---|---|
| 8 | CIRBP | cold-inducible RNA-binding protein | 0.22302 | 0.27538 | 0.28091 | 0.25621 |
| 8 | SMF | metal iron transporter | 0.22208 | 0.25788 | 0.28092 | 0.27461 |
| 8 | | Flavonol synthase/flavanone 3-hydroxylase | 0.22060 | 0.26175 | 0.27134 | 0.28176 |
| 8 | | Fructose-1-6-bisphosphatase | 0.21977 | 0.27389 | 0.27412 | 0.26771 |
| 8 | | Uncharacterized protein | 0.21638 | 0.28786 | 0.26356 | 0.26729 |
| 8 | | 1-aminocyclopropane-1-carboxylate deaminase [EC:3 | 0.21424 | 0.26093 | 0.27649 | 0.28331 |
| 8 | | Uncharacterized protein | 0.21154 | 0.27095 | 0.28607 | 0.26623 |
| 8 | ACSS3, prpE | propionyl-CoA synthetase [EC:6.2.1.17] | 0.21124 | 0.27225 | 0.26649 | 0.28480 |
| 8 | betA, CHDH | choline dehydrogenase [EC:1.1.99.1] | 0.20490 | 0.27387 | 0.28246 | 0.27307 |
| 8 | HOGA1 | 4-hydroxy-2-oxoglutarate aldolase [EC:4.1.3.16] | 0.20316 | 0.27991 | 0.27469 | 0.27640 |
| 9 | | LCCL domain-containing protein | 0.27315 | 0.18472 | 0.27404 | 0.29997 |
| 9 | | RTA-like protein | 0.25261 | 0.20884 | 0.29042 | 0.28239 |
| 9 | SIDD | fusarinine C synthase | 0.24596 | 0.20034 | 0.24539 | 0.33933 |
| 9 | | Cytochrome P450 52A10 | 0.24287 | 0.17823 | 0.29744 | 0.31133 |
| 9 | | C-14 sterol reductase, putative | 0.23935 | 0.21358 | 0.29894 | 0.28214 |
| 9 | | Uncharacterized protein | 0.22787 | 0.20706 | 0.31105 | 0.28654 |
| 9 | | Uncharacterized protein | 0.22150 | 0.22942 | 0.27274 | 0.31015 |
| 9 | | Major facilitator superfamily transporter | 0.22113 | 0.22724 | 0.29197 | 0.29361 |
| 9 | ERG4 | Ergosterol biosynthesis ERG4/ERG24 family protein | 0.22043 | 0.20447 | 0.29810 | 0.30870 |
| 9 | | Autophagy-related protein | 0.20530 | 0.22901 | 0.29603 | 0.30220 |
| 9 | bglX | beta-glucosidase [EC:3.2.1.21]; Cel3e-like protein | 0.20005 | 0.22260 | 0.29106 | 0.31754 |
| 9 | | Uncharacterized protein | 0.19913 | 0.21339 | 0.23833 | 0.37562 |
| 9 | | Pyoverdine/dityrosine biosynthesis protein | 0.19766 | 0.18473 | 0.35120 | 0.29216 |
| 9 | | Oxaloacetate acetylhydrolase | 0.19213 | 0.15376 | 0.28672 | 0.38560 |
| 9 | LDH, ldh | L-lactate dehydrogenase [EC:1.1.1.27] | 0.18809 | 0.20911 | 0.28862 | 0.34200 |
| 9 | bglX | beta-glucosidase [EC:3.2.1.21] | 0.18651 | 0.22701 | 0.29685 | 0.31950 |
| 9 | | Major facilitator superfamily transporter | 0.18371 | 0.23665 | 0.27331 | 0.33583 |
| 9 | | Uncharacterized protein | 0.17294 | 0.19473 | 0.28141 | 0.37344 |
| 9 | CYP56C1 | Cytochrome P450 CYP56C1 | 0.16521 | 0.16713 | 0.36692 | 0.31813 |
| 10 | | Methionine permease | 0.39867 | 0.17094 | 0.32978 | 0.10185 |
| 10 | | High-affinity methionine permease | 0.38468 | 0.13917 | 0.32781 | 0.15783 |
| 10 | | Putative aspartic protease | 0.38045 | 0.15249 | 0.30259 | 0.18125 |
| 10 | | Dibenzothiophene desulfurization enzyme C | 0.37268 | 0.13080 | 0.34009 | 0.16567 |
| 10 | | Alcohol dehydrogenase GroES-like domain-containing | 0.35636 | 0.17657 | 0.30812 | 0.18101 |
| 10 | | Monooxygenase-like protein | 0.35430 | 0.14537 | 0.32370 | 0.19473 |
| 10 | TPP1, CLN2 | Protease S8 tripeptidyl peptidase I (Cln2) | 0.35320 | 0.16804 | 0.29295 | 0.21031 |
| 10 | DUR3 | Urea active transporter | 0.34469 | 0.14437 | 0.34453 | 0.18242 |

| | | | | | | |
|---|---|---|---|---|---|---|
| 10 | E3.1.6.1, aslA | Alkaline phosphatase-like protein | 0.34314 | 0.21085 | 0.30737 | 0.16258 |
| 10 | oxdD | oxalate decarboxylase [EC:4.1.1.2]; Cupin family prote | 0.33262 | 0.20605 | 0.30079 | 0.18840 |
| 10 | | 4-hydroxyacetophenone monooxygenase | 0.33102 | 0.19867 | 0.30265 | 0.19565 |
| 10 | CTNS | Cystinosin/ERS1p | 0.32406 | 0.22479 | 0.28574 | 0.19622 |
| 10 | | TfdA family Taurine catabolism dioxygenase TauD | 0.31212 | 0.18702 | 0.31567 | 0.21395 |
| 11 | | Uncharacterized protein | 0.39696 | 0.27216 | 0.29516 | 0.00000 |
| 11 | | Ferric reductase like transmembrane component | 0.38064 | 0.22400 | 0.35665 | 0.00000 |
| 11 | | Phenol acid carboxylase, putative | 0.36512 | 0.32621 | 0.27432 | 0.00000 |
| 11 | | Conidial wall protein | 0.33592 | 0.26875 | 0.36077 | 0.00000 |
| 11 | | Uncharacterized protein | 0.29953 | 0.32343 | 0.34387 | 0.00000 |
| 11 | RPB12, POLR2K, RPA | DNA-directed RNA polymerases I, II, and III subunit R | 0.25892 | 0.40907 | 0.29517 | 0.00000 |
| 11 | | GNAT family acetyltransferase, putative | 0.00000 | 0.39135 | 0.49303 | 0.00000 |
