# Supplementary material for: Iron homeostasis in the absence of ferricrocin and its consequences in fungal development and insect virulence in Beauveria bassiana
Source: Sci Rep. 2021 Oct 4;11:19624. doi: 10.1038/s41598-021-99030-4 (PMC8490459; doi:10.1038/s41598-021-99030-4)
Supplement: Supplementary file 4 — Supplementary Information 4. [file 41598_2021_99030_MOESM4_ESM.doc]

**Supplemental File S4.** Primers used in targeted gene disruption and their sequences.

| **Primer** | **Sequence (5’-3’)** | **Note** |
| --- | --- | --- |
| *For the ferS disruption* | | |
| FerS-F | GCTCTAGAGCGCTCATCAGTGCCAGTCGTC | *Xba*I sites underlined |
| FerS-R | GCTCTAGAGCGCTGTCAGTGAACGGTG |
| Bar-F | GGAAGATCTTCCGAAAAGTGCCACCTGACGTAG | *Bgl*II sites underlined |
| Bar-R | GGAAGATCTTCCAACAGTTGCGCAGCCTGAATG |
| Upstart_Fp | ACATACCTCCACACGCAACG |  |
| FerS4880_Rp | GCAGGCTCTATCGGTCAGGA |  |
| Bar-100F | AAGCACGGTCAACTTCCGTAC |  |
| Bar-360R | CTTCAGCAGGTGGGTGTAGA |  |
