# Supplementary material for: Iron homeostasis in the absence of ferricrocin and its consequences in fungal development and insect virulence in Beauveria bassiana
Source: Sci Rep. 2021 Oct 4;11:19624. doi: 10.1038/s41598-021-99030-4 (PMC8490459; doi:10.1038/s41598-021-99030-4)
Supplement: Supplementary file 5 — Supplementary Information 5. [file 41598_2021_99030_MOESM5_ESM.pdf]

Supplemental Files S5. Nonribosomal siderophore synthetases used in our phylogenetic analysis and their accession numbers.

Nonribosomal siderophore synthetase	Protein name in phylogenetic tree	Accession number
Aspergillus fumigatus Af293 SidC	AfSidC	XP_753088.1
A. nidulans FGSC A4 SidC	AnSidC	CBF89140.1
Beauveria bassiana BCC 2660 FerS	BbFerS	EJP66049.1
B. bassiana BCC 2660 SidC1	BbSidC1	MZ086759
B. bassiana BCC 2660 SidC2	BbSidC2	MZ086760
B. bassiana BCC 2660 SidC3	BbSidC3	MZ086761
Cochliobolus heterostrophus NPS2	ChNPS2	AAX09984.1
Fusarium graminearum NPS1	FgNPS1	XP_391202.1
F. graminearum NPS2	FgNPS2	XP_385548.1
Magnaporthe oryzae 70-15 SSM1,	MoSSM1	XP_001407762.1
Omphalotus olearius Syn,	OoSyn	AAX49356.1
Schizosaccharomyces pombe Sib1,	SpSib1	CAB72227.1
Ustilago maydis Fer3,	UmFer3	XP_757581.1
U. maydis Sid2	UmSid2	AAB93493.1
